# CNN-Based Device-Agnostic Feature Extraction From ONH OCT Scans

**DOI:** 10.1167/tvst.13.12.5

**Published:** 2024-12-03

**Authors:** Sjoerd J. Driessen, Karin A. van Garderen, Danilo Andrade De Jesus, Luisa Sanchez Brea, João Barbosa-Breda, Bart Liefers, Hans G. Lemij, Doreen Nelson-Ayifah, Angelina Ampong, Pieter W. M. Bonnemaijer, Alberta A. H. J. Thiadens, Caroline C. W. Klaver

**Affiliations:** 1 Department of Ophthalmology, Erasmus Medical Center, Rotterdam, The Netherlands; 2 Department of Epidemiology, Erasmus Medical Center, Rotterdam, The Netherlands; 3 Rotterdam Ophthalmic Institute, Rotterdam Eye Hospital, Rotterdam, The Netherlands; 4 Eye Image Analysis Group Rotterdam, Department of Ophthalmology, Erasmus Medical Center, Rotterdam, The Netherlands; 5 Eye Image Analysis Group Rotterdam, Department of Radiology & Nuclear Medicine, Erasmus Medical Center, Rotterdam, The Netherlands; 6 Eye Image Analysis Group Rotterdam, Rotterdam Ophthalmic Institute, Rotterdam Eye Hospital, Rotterdam, The Netherlands; 7 Research Group Ophthalmology, Department of Neurosciences, KU Leuven, Leuven, Belgium; 8 Cardiovascular R&D Center–UnICatRISE, Faculty of Medicine of the University of Porto, Porto, Portugal; 9 Department of Ophthalmology, Centro Hospitalar e Universitário São João, Porto, Portugal; 10 Glaucoma Service, Rotterdam Eye Hospital, Rotterdam, The Netherlands; 11 Department of Ophthalmology, Komfo Anokye Teaching Hospital, Kumasi, Ghana; 12 Kwame Nkrumah University of Science and Technology, Kumasi, Ghana; 13 Department of Ophthalmology, Radboud University Medical Center, Nijmegen, The Netherlands; 14 Institute for Molecular and Clinical Ophthalmology, Basel, Switzerland

**Keywords:** optic nerve head, OCT, artificial intelligence

## Abstract

**Purpose:**

Optical coherence tomography (OCT)-derived measurements of the optic nerve head (ONH) from different devices are not interchangeable. This poses challenges to patient follow-up and collaborative studies. Here, we present a device-agnostic method for the extraction of OCT biomarkers using artificial intelligence.

**Methods:**

ONH-centered OCT volumes from the Heidelberg SPECTRALIS, ZEISS CIRRUS HD-OCT 5000, and Topcon 3D OCT-1000 Mark I/II and 3D OCT-2000 devices were annotated by trained graders. A convolutional neural network (CNN) was trained on these segmented B-scans and utilized to obtain several ONH biomarkers, such as the retinal nerve fiber layer (RNFL) and the minimal rim width (MRW). The CNN results were compared between different devices and to the manufacturer-reported values using an independent test set.

**Results:**

The intraclass correlation coefficient (ICC) for the circumpapillary retinal nerve fiber layer (cpRNFL) at 3.4 mm reported by the CIRRUS and 3D OCT-2000 was 0.590 (95% confidence interval [CI], –0.079 to 0.901), and our CNN resulted in a cpRNFL ICC of 0.667 (95% CI, –0.035 to 0.939). The cpRNFL at 3.5 mm on the CIRRUS, 3D OCT-2000, and SPECTRALIS generated by the CNN resulted in an ICC of 0.656 (95% CI, 0.055–0.922). Comparing the global mean MRWs from the SPECTRALIS between CNN and manufacturer yielded an ICC of 0.983 (95% CI, 0.917–0.997). The CNN ICC for the MRW among the CIRRUS, 3D OCT-2000, and SPECTRALIS was 0.917 (95% CI, 0.947–0.981).

**Conclusions:**

Our device-agnostic feature extraction from ONH OCT scans showed a higher reliability than the measures generated by the manufacturers for cpRNFL. MRW measurements compared very well among the manufacturers.

**Translational Relevance:**

This open-source software can robustly extract a wide range of biomarkers from any OCT device, removing the dependency on manufacturer-specific algorithms, which has significant implications for patient follow-up and collaborative research.

## Introduction

Optical coherence tomography (OCT) of the optic nerve head (ONH) is used to visualize and measure the anatomical characteristics of the optic disc and surrounding retinal layers. It is used primarily for the diagnosis and monitoring of optic neuropathies, which can be caused by several diseases.^1^^,^^2^ Glaucomatous optic neuropathy (GON) is the most common form, affecting an estimated 80 million people worldwide.^3^ From a histological perspective, optic neuropathies are characterized by the degeneration of retinal ganglion cells and the loss of their axons that make up the retinal nerve fiber layer (RNFL).^4^ This leads to thinning of the RNFL and the ganglion cell layer (GCL) and, in GON, to reduction of the neuroretinal rim and deepening and enlargement of the cup. OCT manufacturers provide dedicated software to quantify these structural changes and aid in the interpretation of ONH OCT which has become an indispensable tool in ophthalmological care.^5^^,^^6^

OCT devices from different manufacturers have similar but not identical approaches to image acquisition and measurement of anatomical parameters. Although they do not seem to differ in their ability to detect glaucoma,^7^^,^^8^ a direct comparison between measurements from devices produced by different manufacturers is not possible.^9^^–^^13^ This can be problematic for patient follow-up in case of referrals from a different center, when changing to a new OCT device, or in large collaborative or multicenter studies. Hence, there is a need for a device-agnostic approach that can be used on OCT images from different manufacturers and provide comparable results. Moreover, such an algorithm would facilitate the possibility to understand whether discrepancies in measurements among devices are primarily due to variation in segmentation and measurement software or to differences in the image acquisition process.

The goal of this study was to develop an algorithm that automatically segments and extracts a number of comparable biomarkers from ONH OCT volumes from different manufacturers. To achieve this, we developed a method to delineate relevant anatomical structures on OCT B-scans using a convolutional neural network (CNN) and an algorithm to compute relevant markers from the full OCT volume. This approach was validated in an independent test set of study participants who were scanned on OCT devices from three different manufacturers.

## Methods

### Image Acquisition

A set of OCT B-scans was randomly selected from databases with OCT volumes that were collected at three study sites from three different countries. Scans were acquired on a Heidelberg SPECTRALIS OCT system (Heidelberg Engineering, Heidelberg, Germany), a ZEISS CIRRUS HD-OCT 5000 (Carl Zeiss Meditec, Dublin, CA, USA), and Topcon 3D OCT-1000 Mark II and 3D OCT-2000 devices (Topcon Corporation, Tokyo, Japan). The CIRRUS OCT was located at the eye clinic of the Komfo Anokye Teaching Hospital in Kumasi, Ghana. The selected scans came from study participants who were enrolled in the Genetics in Glaucoma Patients of African Descent (GIGA) study between May 2015 and January 2021. It is a case–control study investigating genetic, anatomic and environmental factors in a Sub-Sahara African population of primary open-angle glaucoma (POAG) patients and unaffected control participants. Volumetric images centered at the optic disc were acquired by using the standard CIRRUS 6 × 6-mm cube acquisition protocol (200 × 200 axial scans). Both Topcon devices were located at the Rotterdam Study site, where a population-based cohort study is being performed to investigate determinants of age-related diseases in an adult population.^14^ The scans were taken between November 2007 and May 2016. The optic disc–centered volumes were acquired by using the 6 × 6-mm raster scanning protocol (128 × 512 axial scans) on both the OCT-1000 Mark II and OCT-2000 devices. The SPECTRALIS OCT was located at the public hospital Centro Hospitalar e Universitário São João, in Porto, Portugal, where data were collected from patients with POAG and healthy controls between November 2022 and March 2023. The SPECTRALIS images consisted of 24 radial scans and three circular scans at 3.5-mm, 4.1-mm, and 4.7-mm diameter from the center of the ONH.

An additional set of eight healthy eyes from four participants were scanned on the CIRRUS, SPECTRALIS, and 3D OCT-2000 devices to serve as a test set. These consecutive scans were acquired within a 10-month period. The CIRRUS and 3D OCT-2000 devices used for the test set were the same as those used for image acquisition described previously. The SPECTRALIS scanner used for the test set was located at the ophthalmology department of the Erasmus Medical Center in Rotterdam. The CNN and feature extraction algorithm can be accessed at https://github.com/Eyened/OCT-ONH; access to the test set can be requested by contacting the corresponding author.

### Ethical Statements

The Medical Ethics Committee of Erasmus MC (registration no. MEC 02.1015) and the Dutch Ministry of Health, Welfare, and Sport (Population Screening Act WBO, license no. 1071272-159521-PG) approved the use of the individual-level data in the Rotterdam study (RS) participants. The RS is entered into the Dutch National Trial Register (NTR; www.onderzoekmetmensen.nl) and the WHO International Clinical Trials Registry Platform (ICTRP; www.who.int/clinical-trials-registry-platform) under shared catalog number NTR6831. All participants provided written informed consent following the Declaration of Helsinki to participate in the study and to have their information obtained from their treating physicians.

Institutional ethical approval for GIGA study participants was obtained from the Committee for Human Research Publication and Ethics under the School of Medical Sciences of the Kwame Nkrumah University of Science and Technology, Kumasi, Ghana (reference no. CHRPE/AP/540/17) before commencement of the study. Written consent was obtained from all study participants. This study was conducted according to the tenets of the Declaration of Helsinki.

The participation of participants from Porto, Portugal, was approved by the Ethics Review Board of Centro Hospitalar Universitário São João (registration no. CE 112/2023). Informed consent was waived because this was a retrospective data collection with no patient identifiable information. A data transfer agreement was signed between Centro Hospitalar Universitário São João and Erasmus MC.

### Manual Annotation

The randomly selected scans from all devices were manually annotated according to a standardized protocol (see Supplementary Material) by four trained graders from the EyeNED Reading Center at the Erasmus Medical Center in Rotterdam.^15^ The graders were able to adjust the contrast and brightness of the data and to visualize the en face images while annotating each individual B-scan. Graders first assessed general image quality, the presence of movement artifacts within or outside the ONH area, and the position of the ONH on the *x*, *y*, and *z* axes to judge the overall gradability of a volume. If the borders between anatomical structures of interest for annotation were not discernable or if major movement artifacts or positioning of the acquired OCT volume impeded visibility of the ONH or surrounding structures due to misalignment, then that OCT volume would be considered ungradable. The ONH margins were delineated on the en face images, as defined by the margins of Bruch's membrane opening (BMO). In cases of severe gamma-zone peripapillary atrophy (PPA), the ONH margins were annotated on the edge of the scleral flange.

Subsequent annotations were performed on five B-scans from each OCT volume. For the raster scans from the CIRRUS and 3D OCT, the B-scan with the widest BMO was visually selected for annotation. Two B-scans superior and inferior to this point were randomly selected for annotation, ensuring that scans within but also outside of the ONH area were annotated to create a diverse training set. For the SPECTRALIS volumes, approximate horizontal and vertical radial scans were annotated and two randomly chosen diagonal scans in between. The fifth scan was randomly chosen from one of the three circular B-scans.

For each B-scan, the entire RNFL was segmented by highlighting the area between the inner limiting membrane (ILM) and the GCL. Retinal blood vessels were also annotated, to enable future differentiation between RNFL thickness irrespective of vessel presence. Following the neuroretinal tissue past the BMO, it becomes more difficult to discern the border between the RNFL and other layers. Thus, the RNFL border was highlighted until no further signal was visible or until the lamina cribrosa (LC) was reached, at which point the tissue was identified as prelaminar tissue. When identifiable, the LC anterior border was delineated. The axial pixel size of the OCT devices ranged from 2.6 to 3.87 µm,^16^ whereas Bruch's membrane (BM) can be as thin as 2 µm.^17^ Therefore, it was annotated together with the retinal pigment epithelium (RPE) as the RPE–BM complex. If present, alpha-, beta-, and gamma-zone PPA was also annotated.

### Consensus Grading

The four graders discussed B-scans in case of uncertainty regarding annotations. After the first iteration of annotations for the training set was finished, all graded B-scans were checked by a clinician–researcher (SJD). A collection of doubtful annotations was showcased and discussed during two meetings with three ophthalmologists (HL, JBB, PB) and three artificial intelligence specialists (BL, DADJ, LSB) to obtain consensus. Systematic errors and inconsistencies were used to improve the annotation protocol and were communicated to the graders, and faulty annotations were corrected. The test set was annotated by each grader separately and without consultation between graders or with the panel of experts.

### Automatic Segmentation Model

**Figure 1.**
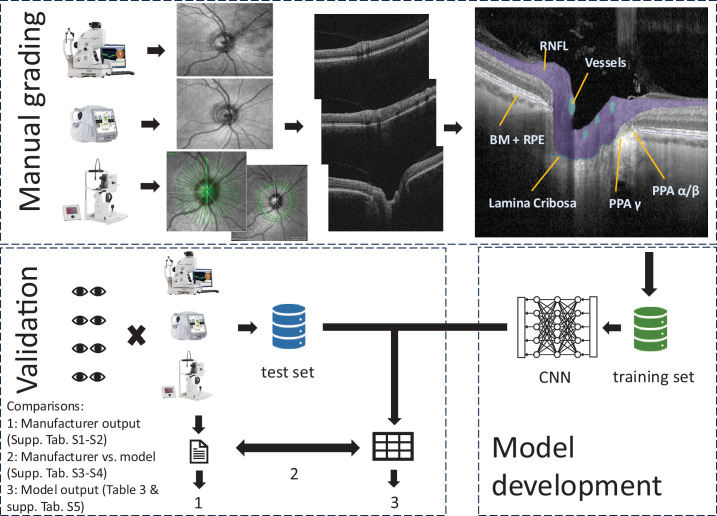
Graphical summary of study workflow: manual annotation, model development, and validation.

The publicly available nnUNetv2^18^ CNN was trained to automatically segment the anatomic structures on OCT B-scans by using manual annotations. Training was performed in fivefold cross-validation on 1030 B-scans using the default settings. The final model is an ensemble of the five cross-validation folds. A graphical summary of the model training and validation is shown in Figure 1.

### Biomarker Extraction

**Figure 2.**
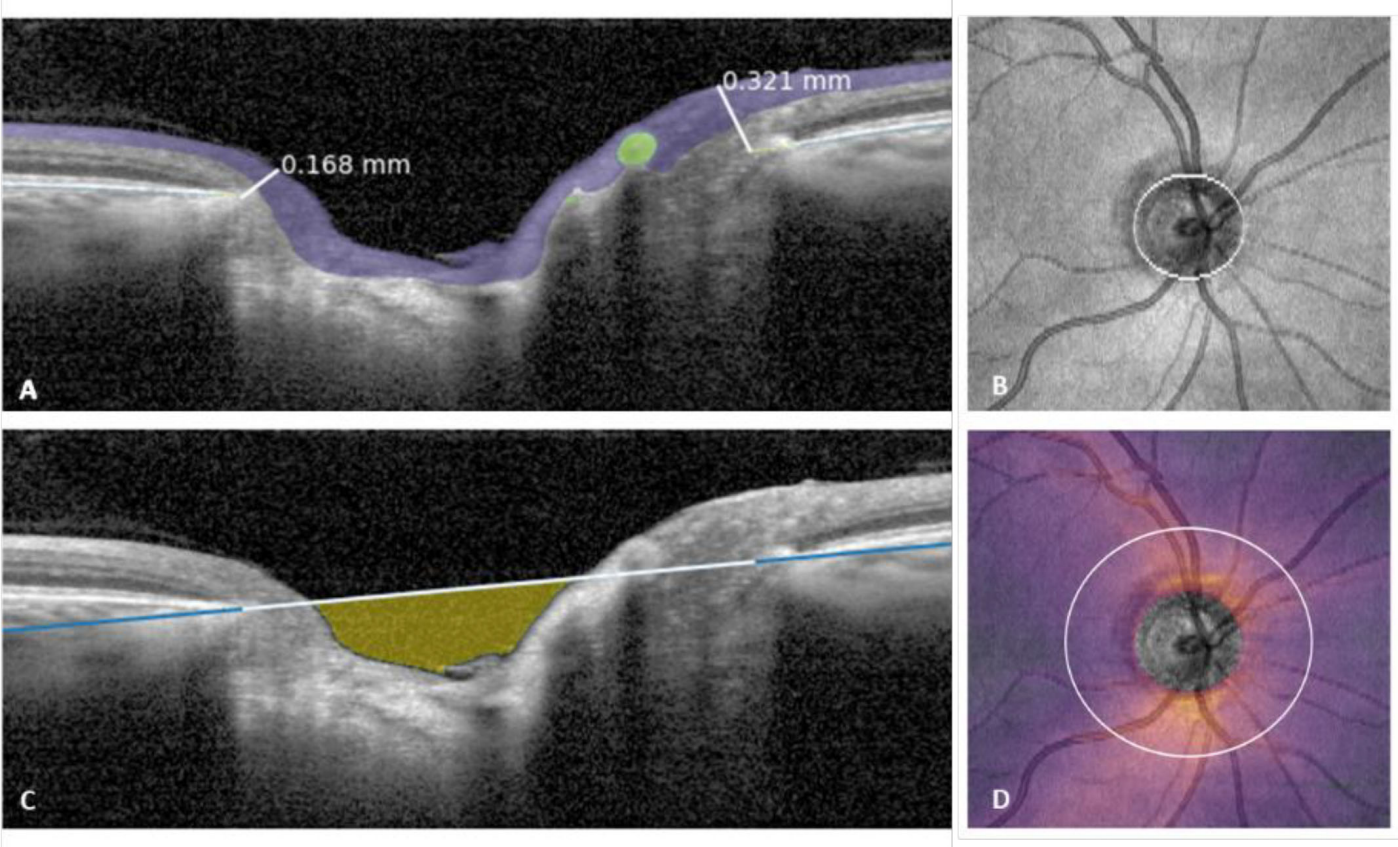
Visualization of extracted biomarkers. (**A**) Minimum rim width, defined as the minimum distance between the BM edge and ILM (*white lines*). (**B**) Surface of the BMO. (**C**) Cup volume, defined as the space between the RNFL/prelaminar tissue and the line that cuts the BM edges (in this instance only visualized in a single B-scan). (**D**) cpRNFL thickness, computable at any diameter from the center of the ONH from cubic acquisition OCT volumes.

The CNN was used on all B-scans to produce a full segmentation of the OCT volumes. As the scan pattern was different between the OCT-2000 and CIRRUS (cube) versus the SPECTRALIS (radial + circular) devices, the biomarker extraction was designed to be as similar as possible. The following biomarkers were extracted from the full segmentations (see Fig. 2):

1. Bruch's membrane opening area (Fig. 2B)—The BMO was defined by the edges of the BM segmentation, including alpha- and beta-zone PPA. To correct minor errors, a morphological closing was applied to the en face projection of the BM and an ellipse was fitted to the resulting boundary points.
2. cpRNFL thickness (Fig. 2D)—The RNFL thickness was computed for each A-scan. To account for vessels interrupting the RNFL, pixels annotated as vessels were considered either part of the RNFL or background, depending on which was the nearest neighboring annotation. On cube acquisitions, the center of the BMO was used as a center point to extract cpRNFL at different diameters (3.4 or 3.5 mm). The mean cpRNFL was computed in clock hours and temporal, superior, nasal, inferior, temporal (TSNIT) sectors (temporal superior, 40°; nasal superior, 40°; nasal, 110°; nasal inferior, 40°; temporal inferior, 40°; temporal, 90°). For the SPECTRALIS acquisition, the circular scans were used and a correction was applied for the tilt of the optic disc with respect to the fovea to define the TSNIT segments (similar to the manufacturer-reported method). This information was not available for the other devices. The RNFL thickness heatmap (Fig. 2D) was generated for illustrative purposes, by plotting the RNFL thickness from each A-scan of a cube acquisition in an en face projection.
3. Bruch's membrane opening minimum rim width (Fig. 2A)—The BMO-MRW was defined as the minimum distance between the edge of the BM and the ILM. For the SPECTRALIS acquisition, the BMO-MRW was extracted from each radial scan. For the cube acquisitions, the 24 SPECTRALIS-like radial scans were generated using the BMO as center, from which the BMO-MRW was extracted.
4. Cup volume (Fig. 2C)—The cup volume was defined as the space enclosed between the line cutting through the BM endpoints, defined in the two-dimensional space of a single B-scan, and the RNFL/prelaminar tissue segmentation. For the cube acquisitions, the volume was summed across the entire BMO to achieve a three-dimensional volume.

### Statistical Analysis

The segmentation accuracy of the CNN was assessed using the Dice similarity coefficient (DSC) on a B-scan basis. For the training set, the mean DSC was computed on the validation set across the five cross-validation folds. For the test set, the model prediction was compared to the grader annotations, and the grader annotations were compared to each other in one-to-one comparisons which were then averaged as a measure of intergrader agreement.

The performance of the CNN in terms of biomarkers was assessed by comparing (1) the extracted biomarkers from the test set to their manufacturer's counterparts, and (2) the biomarkers extracted by the CNNs from the different devices. These test–retest variability measures were analyzed by means of two-way mixed model, absolute agreement, single measure intraclass correlation coefficients (ICCs) in SPSS Statistics 28 (IBM, Chicago, IL). ICC values less than 0.5, between 0.5 and 0.75, between 0.75 and 0.9, and greater than 0.90 were, respectively, regarded as poor, moderate, good, and excellent reliability.^19^

## Results

Descriptive statistics of the data used for training the CNN can be found in Table 1. All participants scanned with the CIRRUS device in Ghana were of African ancestry, whereas all participants scanned with the SPECTRALIS and Topcon devices were of central or southern European ancestry. A total of 95 B-scans from 19 OCT volumes were excluded during the annotation process due to insufficient signal-to-noise ratios or misalignment of the scan or other major artifacts that made annotations impossible to perform. The remaining training set consisted of a total of 1030 B-scans from 206 OCT volumes. There were some missing data on refractive error (23.5% for CIRRUS; 13.2% for SPECTRALIS) and pseudophakia status (3.03% for CIRRUS), and not all study sites collected axial length measurements (19.23% for the 3D OCT-1000 Mark II; 1.82% for the 3D OCT-2000; not available [NA] for the SPECTRALIS and CIRRUS). Descriptive statistics of the test dataset can be found in Table 2. The participants’ ages ranged from 29 to 55 years, and the median spherical refractive errors were –0.63 and –0.88 diopters for OD and OS, respectively. One of the study participants had high myopia with refractive errors of –9.25 and –8.25 for OD and OS.

**Table 1.** Descriptive Statistics of Training Set

	CIRRUS (*N_eyes_* = 68, *N_B-scans_* = 340)	3D OCT-1000 Mark I (*N_eyes_* = 4, *N_B-scans_* = 20)	3D OCT-1000 Mark II (*N_eyes_* = 26, *N_B-scans_* = 130)	3D OCT-2000 (*N_eyes_* = 55, *N_B-scans_* = 275)	SPECTRALIS (*N_eyes_* = 53, *N_B-scans_* = 265)
Age (y), mean ± SD	62.5 ± 14.9	72.6 ± 10.8	78.9 ± 8.09	78.2 ± 6.59	68.1 ± 15.8
Female, % (*n*)	54.4 (37)	75.0 (3)	65.4 (17)	60.0 (33)	67.9 (36)
Ancestry, % (*n*)					
Central European	0 (0)	100 (4)	100 (26)	100 (55)	0 (0)
Southern European	0 (0)	0 (0)	0 (0)	0 (0)	100 (54)
African	100 (68)	0 (0)	0 (0)	0 (0)	0 (0)
Refractive error					
Spherical (D), median ± IQR	0.38 ± 2.00^a^	2.00 ± 3.00	1.13 ± 2.13	1.38 ± 1.75	0.75 ± 1.50^c^
Cylindrical (D), median ± IQR	−1.00 ± 1.44^a^	−0.75 ± 1.75	−1.00 ± 1.13	−1.13 ± 1.25	−1.00 ± 1.50^c^
Axial length (mm), mean ± SD	NA	NA	23.23 ± 1.00^d^	23.12 ± 0.91^e^	NA
IOP (mmHg), mean ± SD	17.9 ± 5.36	12.8 ± 1.50	13.9 ± 2.19	14.7 ± 3.04	15.3 ± 4.62
Glaucoma, % (*n*)	67.6 (46)	0 (0)	0 (0)	3.6 (2)	52.8 (28)
Cataract, % (*n*)	20.6 (14)	0 (0)	3.8 (1)	5.5 (3)	1.90 (1)
Pseudophakic, % (*n*)	21.2 (14)^b^	25.0 (1)	42.3 (11)	36.4 (20)	52.8 (28)
Retinal pathology					
Epiretinal membrane, % (*n*)	0 (0)	25.0 (1)	19.2 (5)	21.8 (12)	5.7 (3)
Diabetic retinopathy, % (*n*)	0 (0)	0 (0)	0 (0)	0 (0)	1.90 (1)
AMD, % (*n*)	0 (0)	0 (0)	11.5 (3)	1.8 (1)	3.80 (2)
CRVO, % (*n*)	0 (0)	0 (0)	0 (0)	0 (0)	1.9 (1)
Vitelliform retinopathy, % (*n*)	0 (0)	0 (0)	0 (0)	0 (0)	1.9 (1)
DM, % (*n*)	26.5 (18)	25.0 (1)	30.8 (8)	29.1 (16)	35.8 (19)
Hypertension, % (*n*)	61.8 (42)	50.0 (2)	88.5 (23)	96.4 (53)	43.4 (23)

*N_eyes_* for missing cases. AMD, age-related macular degeneration; CRVO, central retinal vein occlusion; DM, diabetes mellitus; IOP, intraocular pressure; IQR, interquartile range; NA, not available; *N_B-scans_*, total number of OCT B-scans used; *N_eyes_*, total number of eyes/OCT volumes used; SD, standard deviation.

a *N_total_* = 52.

b *N_total_* = 66.

c *N_total_* = 46.

d *N_total_* = 21.

e *N_total_* = 54.

**Table 2.** Descriptive Statistics of the Test Set (*N* = 4)

Statistic	Value
Age (y), median (range)	40.5 (29 to 55)
Female, % (*n*)	50 (4)
Ethnicity	
European ancestry, % (*n*)	100 (4)
Refractive error (D)	
OD spherical	−0.63 (−9.25 to –0.25)
OD cylindrical	−0.88 (−1.75 to −0.75)
OS spherical	−0.88 (−8.25 to −0.50)
OS cylindrical	−0.88 (−2.00 to 0.00)
Axial length (mm), median (range)	
OD	24.47 (23.55 to 27.13)
OS	24.68 (23.27 to 26.58)
IOP (mmHg), median (range)	
OD	15.50 (14 to 17)
OS	16.50 (15 to 17)
Glaucoma, % (*n*)	0 (0)
Cataract, % (*n*)	0 (0)
Pseudophakic, % (*n*)	0 (0)

*N* is the total number of participants for whom OCT scans of both eyes were included. D, diopters.

The segmentation accuracy in terms of mean DSC for the validation sets is presented in Supplementary Table S1. The segmentation accuracy of the final model and the concordance between grader segmentation on the test set are presented in Supplementary Table S2. There was poor agreement in the segmentation of the LC and PPA labels (all less than 0.2 DSC). There was a notable difference in performance between the devices for BMO, with relatively high overlap on the SPECTRALIS (SPECTRALIS: 0.92 DSC model vs. graders; 3D OCT-2000: 0.67 DSC model vs. graders; CIRRUS: 0.67 DSC model vs. graders). Marked differences among the devices were also seen for the vessels, with poor overlap on the CIRRUS (0.29 DSC intergrader; 0.18 DSC model vs. graders), relatively higher overlap on the 3D OCT-2000 (0.36 DSC intergrader; 0.37 DSC model vs. graders), and the highest overlap on the SPECTRALIS (0.60 DSC intergrader; 0.58 DSC model vs. graders). The overlap on the RNFL label was comparable among devices, all between 0.75 and 0.85 DSC. In general, the model performance was comparable to the intergrader variation for all labels.

Comparison of cpRNFL and MRW measurements from the CNN output among the manufacturers are presented in Table 3. Comparison of manufacturer proprietary output between the devices can be found in Supplementary Tables S3 and S4. Comparison of the CNN output to the manufacturers’ output can be found in Supplementary Tables S5 and S6. The unavailable (NA) values were a consequence of dissimilar measurement and acquisition methods among the devices. For example, quartiles were reported instead of TSNIT segments, and the circular acquisition protocol of the SPECTRALIS device at fixed diameters did not allow any direct comparisons. Consequently, the cpRNFL thickness was compared at a 3.4- and 3.5-mm diameter, following the measurements provided by the manufacturers.

**Table 3.** Concordance of cpRNFL Thickness and BMO-MRW Measures Comparing CNN Output Among Manufacturers

	CNN						
	CIRRUS vs. 3D OCT-2000		CIRRUS vs. SPECTRALIS		3D OCT-2000 vs. SPECTRALIS		
	ICC (95% CI)	Mean of Absolute Differences ± SD (µm)	ICC (95% CI)	Mean of Absolute Differences ± SD (µm)	ICC (95% CI)	Mean of Absolute Differences ± SD (µm)	CIRRUS vs. 3D OCT-2000 vs. SPECTRALIS ICC (95% CI)
cpRNFL at 3.4 mm	0.667 (−0.035 to 0.939)	8.39 ± 2.47	NA	NA	NA	NA	NA
cpRNFL at 3.5 mm	0.684 (−0.037 to 0.943)	7.90 ± 2.41	0.483 (−0.046 to 0.878)	11.10 ± 3.63	0.896 (0.203 to 0.982)	3.19 ± 2.74	0.656 (0.055 to 0.992)
Temporal superior	0.626 (−0.013 to 0.910)	8.30 ± 9.06	0.532 (−0.196 to 0.884)	10.52 ± 8.99	0.866 (0.457 to 0.972)	7.11 ± 5.46	0.702 (0.327 to 0.923)
Nasal superior	0.716 (−0.079 to 0.944)	13.60 ± 9.91	0.683 (−0.088 to 0.934)	13.41 ± 10.03	0.966 (0.839 to 0.993)	5.21 ± 2.68	0.794 (0.308 to 0.954)
Nasal	0.840 (0.011 to 0.972)	8.13 ± 6.10	0.706 (−0.045 to 0.948)	12.90 ± 4.41	0.937 (0.477 to 0.989)	4.84 ± 4.62	0.830 (0.243 to 0.966)
Inferior nasal	0.826 (0.025 to 0.968)	13.38 ± 10.71	0.740 (−0.081 to 0.951)	18.27 ± 11.71	0.970 (0.784 to 0.994)	6.85 ± 2.95	0.848 (0.367 to 0.969)
Inferior temporal	0.685 (−0.095 to 0.936)	11.46 ± 7.77	0.479 (−0.118 to 0.867)	16.84 ± 10.61	0.903 (0.314 to 0.982)	6.59 ± 3.22	0.667 (0.123 to 0.920)
Temporal	0.277 (−0.509 to 0.799)	8.01 ± 5.68	0.034 (−0.641 to 0.684)	12.16 ± 6.89	0.909 (0.635 to 0.981)	4.16 ± 2.35	0.464 (0.034 to 0.837)
BMO-MRW, mean	0.899 (0.573 to 0.979)	23.40 ± 20.59	0.860 (0.467 to 0.970)	25.12 ± 29.38	0.974 (0.070 to 0.997)	17.84 ± 6.13	0.919 (0.757 to 0.981)
Temporal-superior	0.994 (0.698 to 0.698)	19.28 ± 18.68	0.940 (0.727 to 0.988)	22.51 ± 14.66	0.940 (0.682 to 0.988)	16.98 ± 21.95	0.941 (0.821 to 0.987)
Nasal superior	0.903 (0.584 to 0.980)	21.19 ± 27.67	0.960 (0.811 to 0.992)	18.85 ± 8.61	0.947 (0.410 to 0.991)	18.83 ± 13.59	0.935 (0.793 to 0.986)
Nasal	0.614 (−0.004 to 0.905)	38.35 ± 53.95	0.489 (−0.121 to 0.862)	59.49 ± 69.98	0.960 (0.519 to 0.993)	23.59 ± 17.37	0.725 (0.363 to 0.929)
Inferior nasal	0.644 (−0.031 to 0.917)	52.94 ± 48.66	0.583 (−0.037 to 0.896)	58.29 ± 65.61	0.963 (0.590 to 0.994)	24.96 ± 20.27	0.775 (0.458 to 0.944)
Inferior temporal	0.885 (0.429 to 0.977)	22.64 ± 25.47	0.957 (0.815 to 0.991)	13.00 ± 9.44	0.842 (0.145 to 0.970)	33.33 ± 19.65	0.886 (0.606 to 0.975)
Temporal	0.952 (0.776 to 0.990)	18.78 ± 11.21	0.943 (0.743 to 0.988)	21.18 ± 12.84	0.978 (0.848 to 0.996)	11.99 ± 11.57	0.959 (0.875 to 0.991)

Agreement between the CIRRUS and 3D OCT-2000 proprietary device output for mean cpRNFL thickness at a 3.4-mm diameter around the ONH was moderate (ICC = 0.590; 95% CI, –0.079 to 0.901). The same comparison performed with CNN-extracted measurements indicated a modest increase in agreement (ICC = 0.667; 95% CI, –0.035 to 0.939). All three devices could be compared by using CNN-generated cpRNFL measurements at a 3.5-mm diameter. This resulted in a moderate agreement (ICC = 0.656; 95% CI, 0.055–0.992). In two-way comparisons, the best agreement was achieved between the SPECTRALIS and 3D OCT-2000 (ICC = 0.896; 95% CI, 0.203–0.982), whereas the CIRRUS and SPECTRALIS had the worst agreement (ICC = 0.483; 95% CI, –0.046 to 0.878). Directly comparing the manufacturers’ mean cpRNFL thickness to the CNN-generated mean cpRNFL thickness resulted in good agreement for the CIRRUS (ICC = 0.815; 95% CI, 0.301–0.960]), 3D OCT-2000 (ICC = 0.916; 95% CI, 0.668–0.982), and SPECTRALIS (ICC = 0.873; 95% CI, –0.038 to 0.981).

The CIRRUS and 3D OCT-2000 do not provide MRW measurements and could therefore not be compared. The SPECTRALIS proprietary output compared to the CNN output for mean MRW was excellent (ICC = 0.983; 95% CI, 0.917–0.997). Agreement on mean MRWs from the CNN among the three devices was also excellent (ICC = 0.919; 95% CI, 0.757–0.981).

Agreement on cup volumes compared between the CIRRUS and 3D OCT-2000 manufacturer output (ICC = 0.993; 95% CI, 0.923–0.999) and CNN output (ICC = 0.990; 95% CI, 0.950–0.989) (Supplementary Table S7) was excellent. Comparing manufacturer output to CNN output yielded moderate agreement for the CIRRUS (ICC = 0.680; 95% CI, 0.019–0.927) and 3D OCT-2000 (ICC = 0.713; 95% CI, 0.088–0.935), although the manufacturers’ definitions of cup volume differed from that used for the CNN. CNN output for alpha-, beta-, gamma PPA and the LC was not satisfactory and therefore was not included in further comparison analyses. Additional elaboration of this point can be found in the Discussion section.

### Visualization of Results

**Figure 3.**
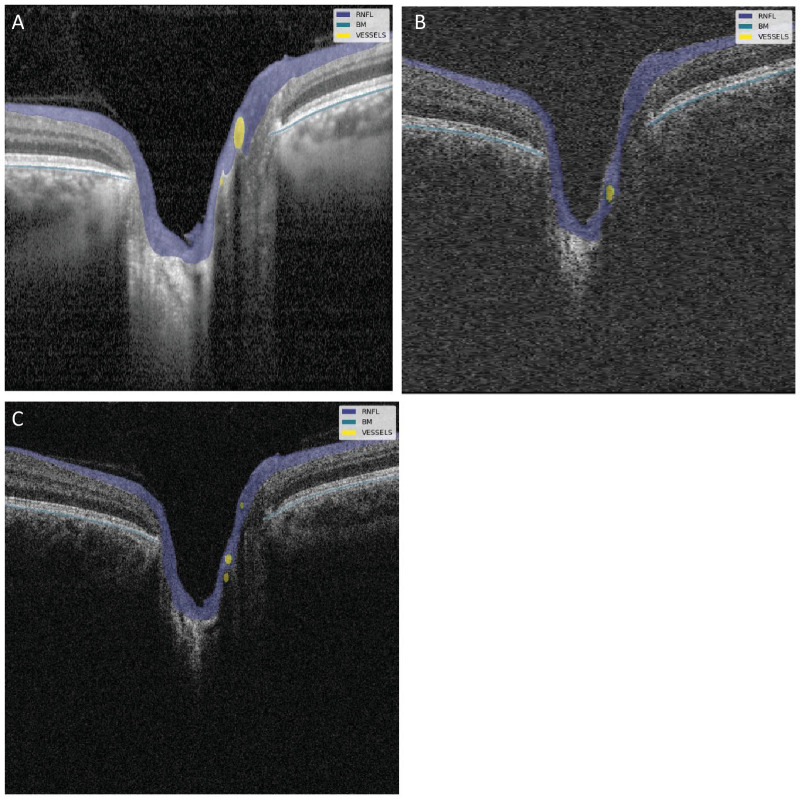
Visualization of CNN model results for one of the eyes in the test set. The annotation of the RNFL (*purple*), BM (*blue*), and vessels (*yellow*) is overlaid on the central B-scan from the three different devices: (**A**) SPECTRALIS, (**B**) CIRRUS, (**C**) 3D OCT-2000.

**Figure 4.**
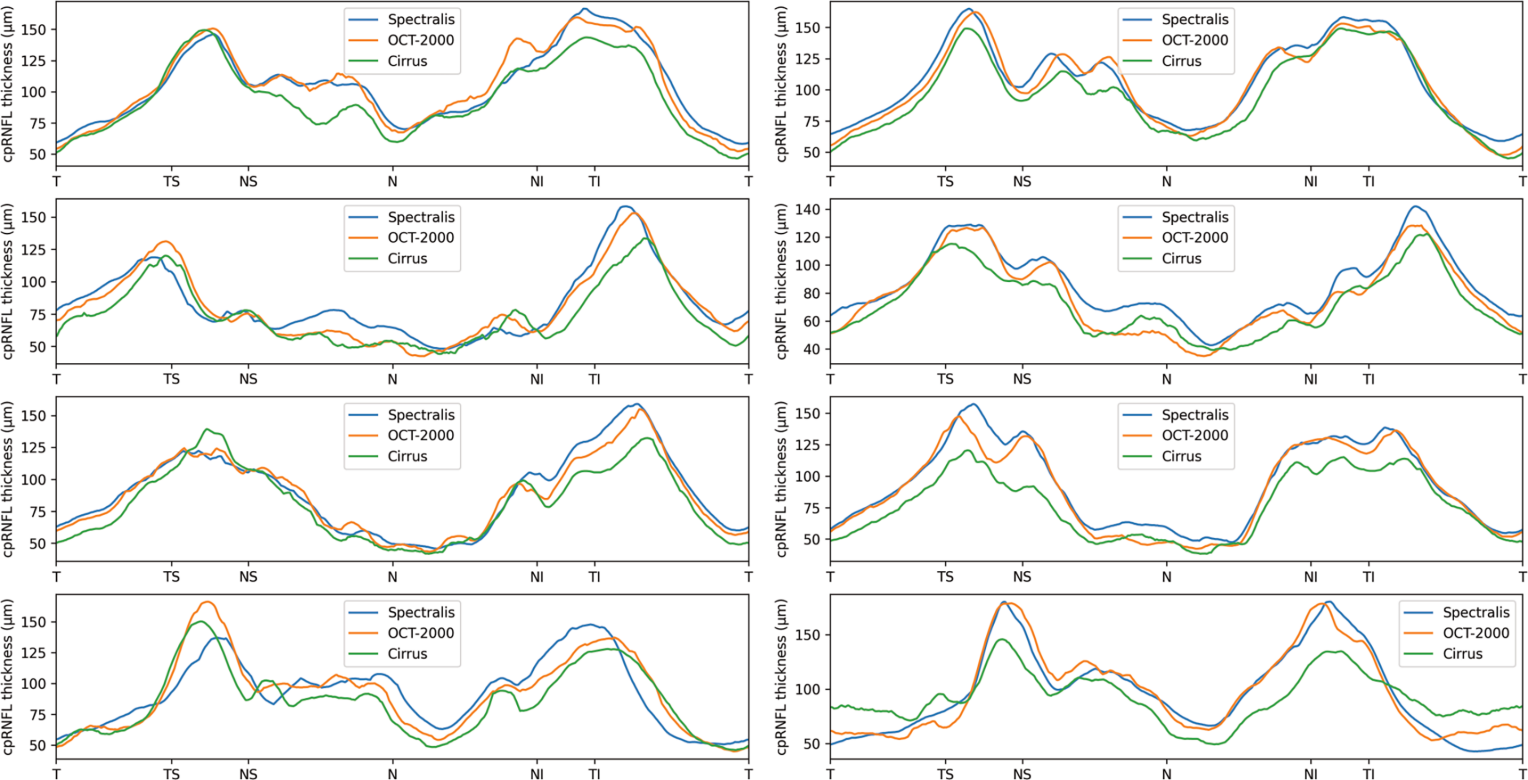
cpRNFL measurements by model for each of the devices in all eight eyes (*left*, OD; *right*, OS) from the test set.

**Figure 5.**
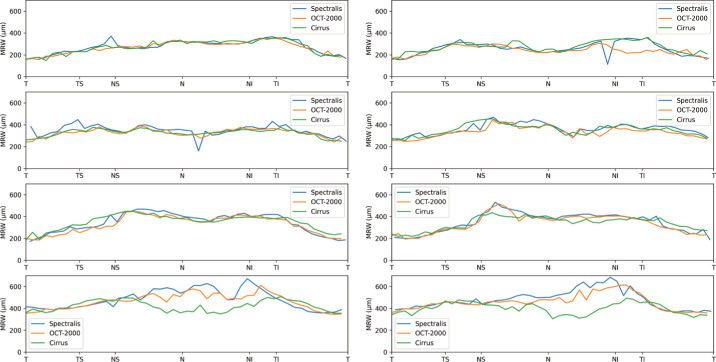
MRW measurements by model for each of the devices in all eight eyes (*left*, OD; *right*, OS) from the test set.

**Figure 6.**
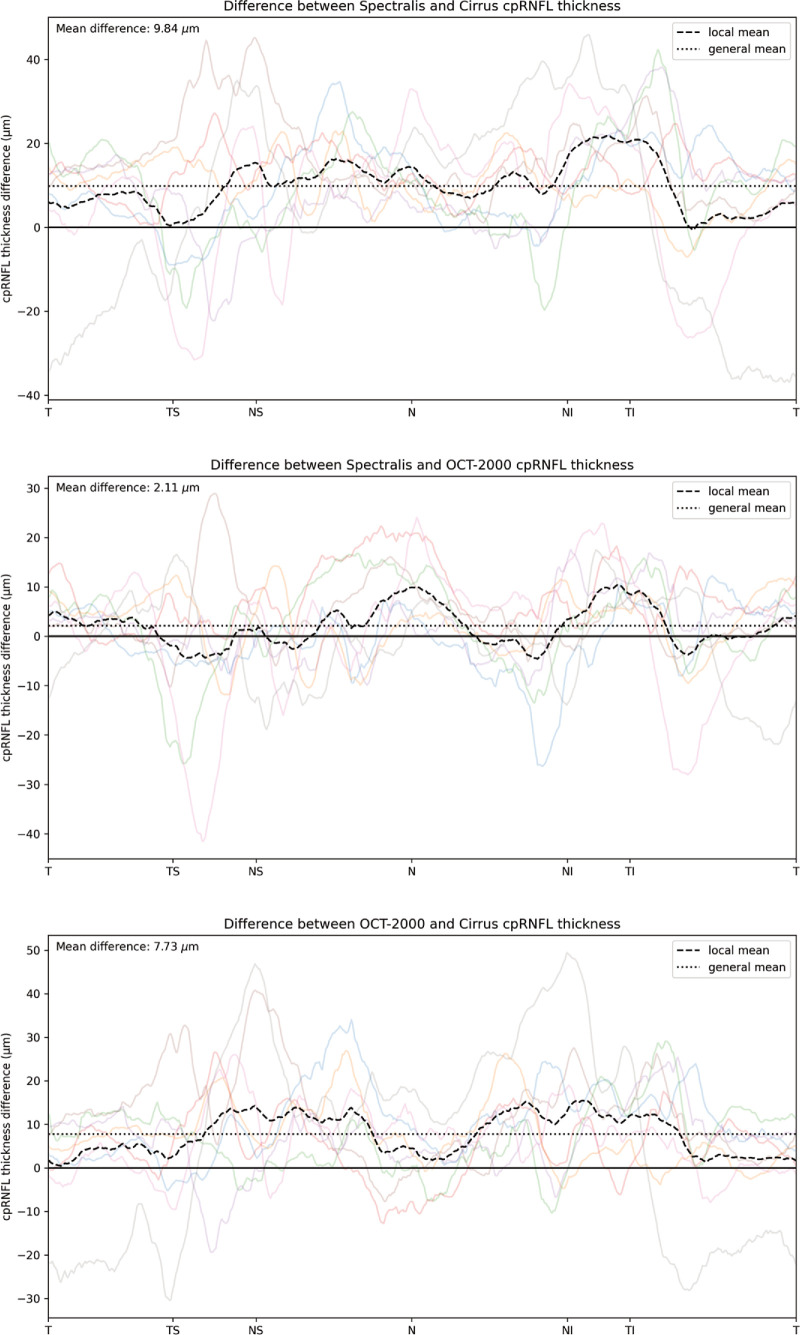
Visualization of the cpRNFL mean differences for the model output among the different devices. The individual differences for all eight eyes are plotted in the background, with the mean of those differences across the circular scan shown as a *dashed black line*, the general mean as a *dotted* *line*, and the zero difference as a *solid black line*.

**Figure 7.**
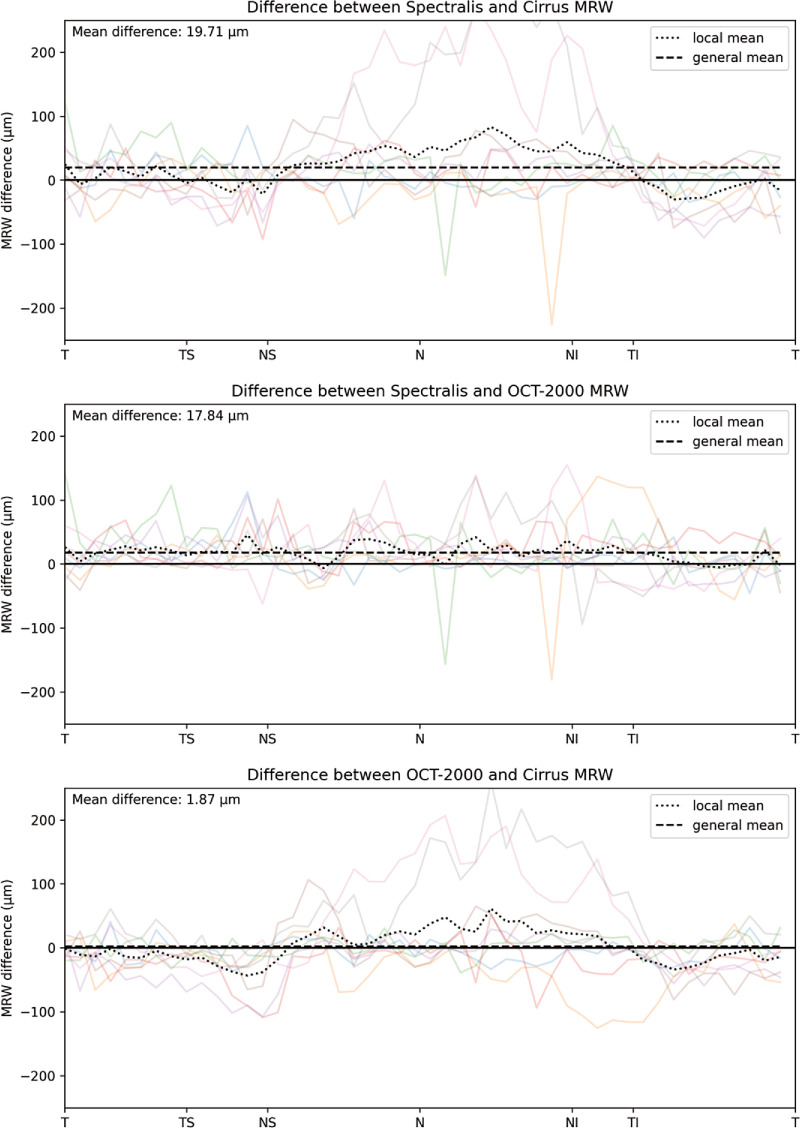
Visualization of the MRW mean differences for the model output among the different devices. The individual differences for all eight eyes are plotted in the background, with the mean of those differences across the circular scan shown as a *dashed black line*, the general mean as a *dotted* *line*, and the zero difference as a *solid black line*.

Figure 3 shows an example of the CNN segmentation model output for each of the devices for the central horizontal B-scan (defined by the widest BMO) in one of the eight eyes of the test set. The anatomy of the BM and RNFL is captured well, although the placement of the vessels is not identical for the devices. To make a qualitative interpretation of the differences between extracted features, a number of visualizations were made. The CNN-generated cpRNFL and MRW measurements from the three different devices were plotted together for each eye in the test set (Figs. 4, 5). The aggregated mean difference for model output between devices was also computed across the entire cpRNFL circle (Fig. 6, dashed line). These measurements were smoothed by taking a moving average over a window of 9° to avoid focal effects of the vessels. The thickness measurements from the CIRRUS were generally lower than those of the SPECTRALIS and 3D OCT-2000, with a mean difference of 9.8 µm between the CIRRUS and SPECTRALIS and 2.1 µm between the 3D OCT-2000 and SPECTRALIS. The aggregated mean difference for model output among devices for MRW measurements was also plotted together for each eye in the validation set (Fig. 7). The observed variation of measurement differences among the devices made it inappropriate to calculate a standard correction factor to translate the measurements of one device to the other.

## Discussion

A segmentation model (CNN) and feature extraction algorithm for ONH-related parameters were developed on manually annotated OCT scans from three different manufacturers and tested on eight eyes that were all scanned on these three devices. Comparisons were performed among the CNN-generated measurements of the three different devices, among the manufacturers’ proprietary output from the three devices (when possible), and between the CNN-generated measurements and manufacturers’ proprietary output for each device. MRW measurements, generated with our CNN for the devices that do not provide this parameter, compared very well among the manufacturers. Agreement among the manufacturers’ proprietary cpRNFL measurements was moderate at best, with only slight improvements when comparing the CNN-generated cpRNFL measurements among devices. It should be noted that the cpRNFL measurements from the CNN compared excellently between the SPECTRALIS and 3D OCT-2000, with more variation observed when comparing the CIRRUS to other devices. This suggests that a difference in acquisition technique (a raster scan as implemented by the 3D OCT-2000 vs. a radial and circular scan as implemented by the SPECTRALIS) is not a big factor in image and subsequent measurement variation. Moreover, agreement between the CNN and manufacturer proprietary output for each device was good overall.

There can be multiple sources contributing to variations in cpRNFL measurements. The segmentation is highly dependent on relative image intensities, which can differ between devices, and implementation of the device-specific segmentation algorithm. Another source of variation is the location of the measurements. For example, the localization of the BMO center is essential to achieve consistent measurements. However, variation in the detection of the BMO edge, which can be difficult to see in case of PPA or a large amount of prelaminar tissue which affects signal penetration, can affect the localization of the BMO center. Head tilt can also cause variation in measurements due to a slight change in location of the TSNIT sectors by means of rotation.

We found substantial intergrader variation for many of the labels, which indicates an inherent ambiguity in the images. By comparing the CNN to the graders and to the manufacturers’ output, we aimed to quantify the degree of variation as much as possible. We were not able to compare grader annotations directly to the manufacturers’ output, as those biomarkers are not defined on the basis of individual B-scans. Ultimately, it is very challenging to pinpoint the exact source of interdevice variation and whether an algorithm would be able to improve that.

It is possible that improving the segmentation accuracy would also decrease the interdevice variation. We found that, especially for BM, the performance differed among the devices. An improvement could be achieved by increasing the size of the training dataset or improvements in the model architecture. We selected the nnUNetv2 model architecture, as its performance has been validated on a wide variety of medical image segmentation tasks,^18^ showing state-of-the-art performance overall. Considering that the model performance is similar to the intergrader variation, we do not expect large improvements to be easily achieved.

Our custom feature extraction algorithm is able to harmonize the location of measurements among devices, limited only by the imaging protocol. Improvements of the algorithm may be achieved by enhancing BMO edge detection and implementing correction of head tilt by means of fovea localization, which is a feature already used in the SPECTRALIS software, but not the CIRRUS and 3D OCT-2000 devices.

Structural differences between the devices in terms of cpRNFL thickness were apparent, with the CIRRUS device providing generally thinner measurements. Nevertheless, we did not find a correction factor that could reliably translate the measurements of one device to the other. Especially in the temporal superior and temporal inferior regions, where most of the RNFL tissue and vessel structures are located, differences among the devices were generally the largest. This is likely due to an increased sensitivity to precise location of the measurement, because a slight shift in location due to, for example, head tilt could make a big difference, as well as the ambiguity of the RNFL lower boundary as it intersects with large vessels.

The MRW measurements from the CNN were more consistent among devices than the cpRNFL. The ILM in the BMO region is typically highly discernible, which is not always the case with the border between the RNFL and GCL, and there is no interference of vessel boundaries in its segmentation. However, the accuracy of MRW measurements is primarily limited by the accuracy of the segmentation of BMO edges, which was the biggest source of variation for MRW measurements in our test set. Two eyes from the test set had a smaller BMO size and consequently thicker prelaminar tissue, which resulted in an obscured visibility of the BMO edges on the CIRRUS OCT volume. Similar to the cpRNFL measurements, the highest agreement in MRW measurements was observed between the 3D OCT-2000 and SPECTRALIS volumes.

The LC has been implicated in POAG pathophysiology^20^^–^^23^ and was therefore one of the included anatomical structures in our annotation protocol. However, the visibility of the anterior surface of the LC varied significantly across different OCT devices, and the posterior surface was generally undetectable. The radial scanning protocol with high averaging of images from the SPECTRALIS provided the best discernibility of the LC. Nonetheless, eyes with thicker prelaminar tissue or tiled discs still resulted in poor and sometimes no visibility of the LC. This severely hampered more detailed analyses of this structure with ICCs in our test set.

Although the enhanced depth imaging feature of the SPECTRALIS could potentially improve results,^24^ its absence in other OCT devices rendered its use impractical for the development of a universal, device-agnostic segmentation algorithm. Consequently, the LC was excluded from subsequent analyses in our study.

The alpha-zone PPA is not associated with pathology, but the beta-zone PPA is associated with both the presence and progression of glaucoma.^25^^,^^26^ Gamma-zone PPA is predominantly associated with (high) myopia,^27^ which is of interest in glaucoma research due to the known association between myopia and glaucoma. We therefore included alpha-, beta-, and gamma-PPA in the annotation protocol and subsequent training of the CNN. However, the CNN often did not find gamma-PPA in cases where this was present and made errors in distinguishing alpha- from beta-PPA. Hence, distinctions among categories of PPA were omitted from further analyses because the resulting ICCs would not be informative.

This study has several strengths. To our knowledge, this is the first study to develop a device-agnostic segmentation algorithm for ONH OCT scans with big variation in image quality from patients of different ethnicities and ages. Moreover, the CNN is trained with a large set of manually annotated OCT volumes captured with a variety of devices from different manufacturers. Importantly, we collected a test dataset of eyes that were scanned with all three devices to investigate the robustness and potential weaknesses of our algorithm.

This study also has some limitations. Although we see our test dataset as a major asset of our study, it is limited in sample size. This resulted in relatively wide CIs accompanying the ICCs from our comparative analyses, with single relative outliers having a big impact on the ICC values even though the means of the absolute differences often fell within the reported test–retest variability of some OCT devices.^28^ Another limitation is the lack of pathology in the test set. Poor signal-to-noise ratios, thin tissues of interest, and unwanted eye movement can be a result of pathology and impair the performance of segmentation algorithms. There was also a difference in population characteristics between the training data and the test set, but we did not see a large difference in CNN performance as a result.

There was quite some variation in axial lengths, presence of PPA, and prelaminar tissue thickness in our relatively small test set. These factors could have affected the measurement of ONH-related features. This well represents real-world physiological variation which should be considered when implementing a segmentation algorithm in practice. In this study, only data captured with spectral-domain OCT (SD-OCT) devices were used to train the algorithm. Swept-source OCT (SS-OCT) devices have become more common in clinical practice and offer some advantages over SD-OCT, such as deeper tissue penetration and faster acquisition speeds.^29^ Future improvements of our algorithm would include an even larger variety of training and validation data. Although our algorithm was not validated on SS-OCT images, there are no technical limitations to do this and it can be easily adapted to other devices by adding a small amount of training data to make it even more robust.

The results of this study concern the reproducibility of ONH features across devices in the general population. However, with these results we cannot draw conclusions concerning the relative accuracy of our measurements or those from the manufacturers or their utility in the assessment of (glaucomatous) optic neuropathy. Future research in both healthy populations and glaucoma patients is needed to assess clinical utility. Moreover, future research should also evaluate the performance of this approach between populations of different ancestries.

With this study, we developed a device-agnostic segmentation and feature extraction algorithm for OCT scans of the ONH, with potential value for collaborative research projects and clinical practice. The algorithm allowed us to compare several robust ONH features among different devices while circumventing proprietary segmentation software. Future studies must further evaluate the reproducibility of the algorithm. We have made the trained CNN and the feature extraction algorithm publicly available for research purposes, and the test set is available upon reasonable request.

## Supplementary Material

Supplement 1

Supplement 2
