# Supplementary material for: CNN-Based Device-Agnostic Feature Extraction From ONH OCT Scans
Source: Transl Vis Sci Technol. 2024 Dec 3;13(12):5. doi: 10.1167/tvst.13.12.5 (PMC11620008; doi:10.1167/tvst.13.12.5)

Protocol grading optic nerve OCT

In the viewer, you can zoom in by holding down the shift key and scrolling. You can adjust contrast/brightness by holding down the ctrl key and dragging the mouse (holding the left mouse button). Drag down for more contrast, to the right for more brightness. By right-clicking with the ctrl key or pressing the escape key, you can reset to the default values. With the spacebar, you can temporarily make previously drawn structures invisible. There is an undo button that allows you to cancel the last drawn structure. You can also erase what you have drawn by right-clicking. With the S key, you can automatically select the structure your mouse is pointing at. Alt + scrolling adjusts the size of your brush tool when it is selected. This can also be done with the number keys or the + and - keys.

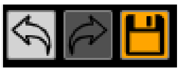

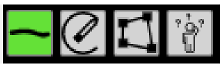

1. Assess the quality of the OCT volume

Be realistic here; this is clinical data and you will certainly encounter OCT volumes where the signal is simply too poor to grade.

- Good
- Moderate
- Not gradable

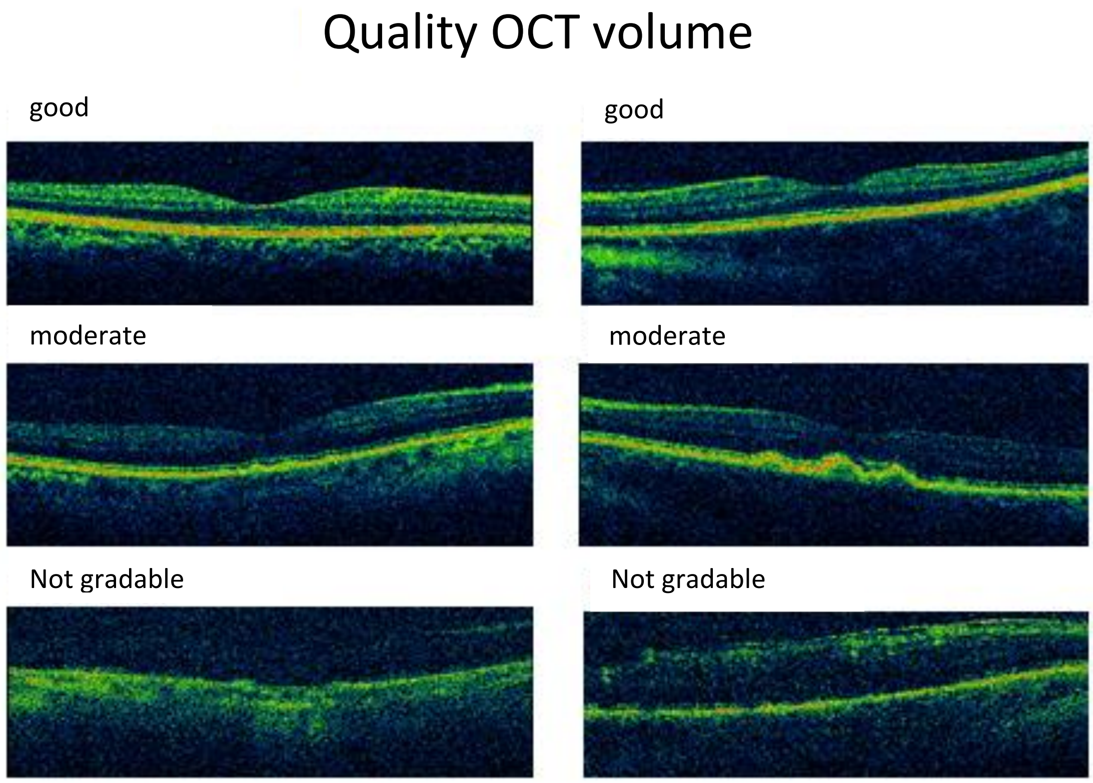

2. Assess the position of the OCT volume

- ○ Good/central
- ○ Not gradable/out of frame

Position OCT volume

Good/central

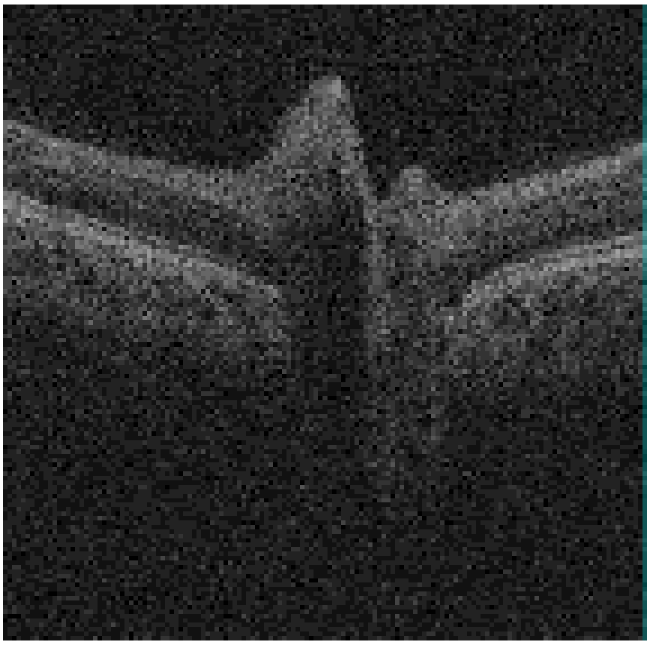

Not gradable/out of frame

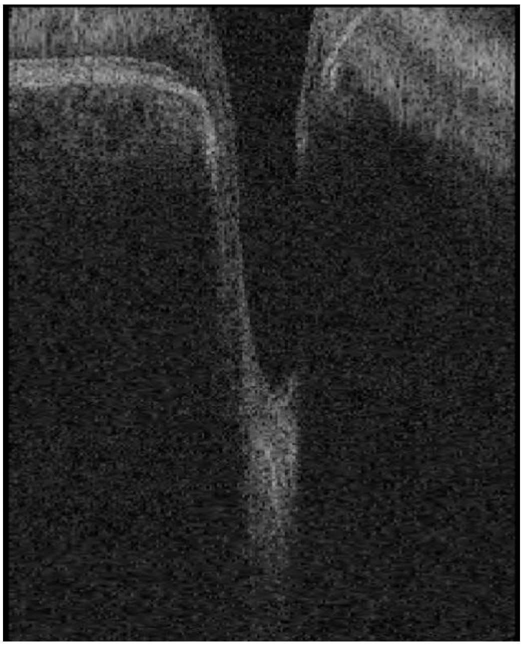

3. Assess for any saccades (if present both outside and inside the optic disc, choose "inside the optic disc")

- ○ good/not present
- ○ outside optic disc
- ○ inside optic disc

Artefacts/saccades

Outside optic disc

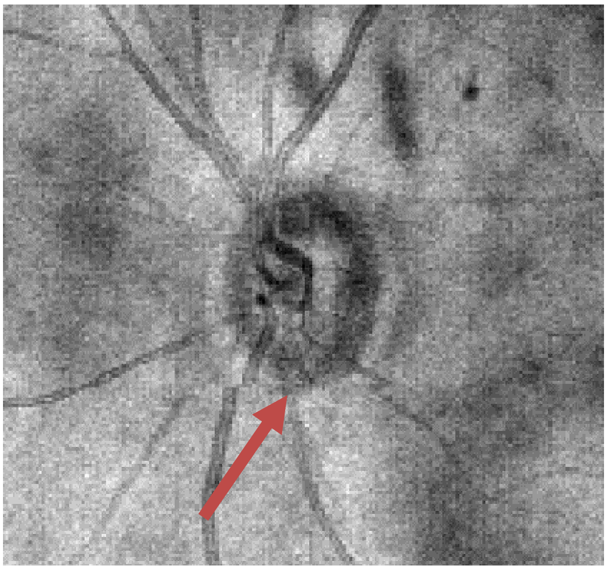

Inside optic disc

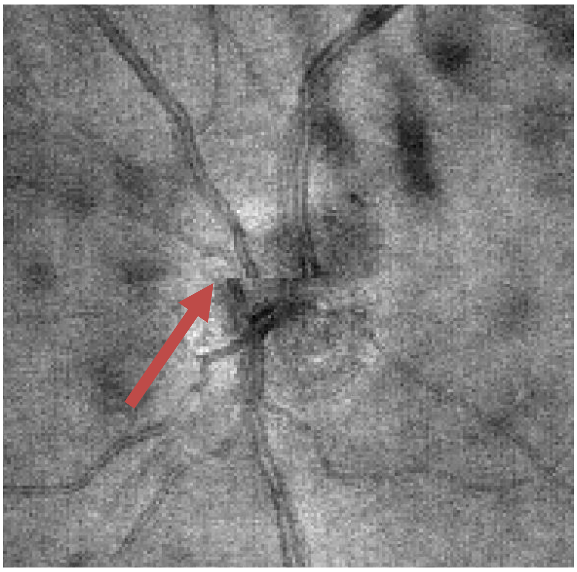

4. Other remarks on quality of OCT scan (optional)

…

5. Disk en-face

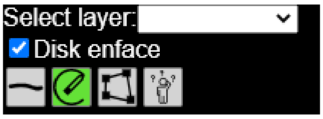

Draw the outline of the entire optic disc on the enface image (left window). Make sure the "Disc enface" box is checked. It is best to use the "outline tool" for this. The outer border of the optic disc outline is typically Bruch's membrane (BM). You can check in the middle window to ensure you are at the correct boundary (red arrow). This boundary can sometimes be difficult to see on the enface image in the case of peri-papillary atrophy. In the case of γ-zone PPA (no RPE and BM), draw the boundary up to the scleral band (see the image below).

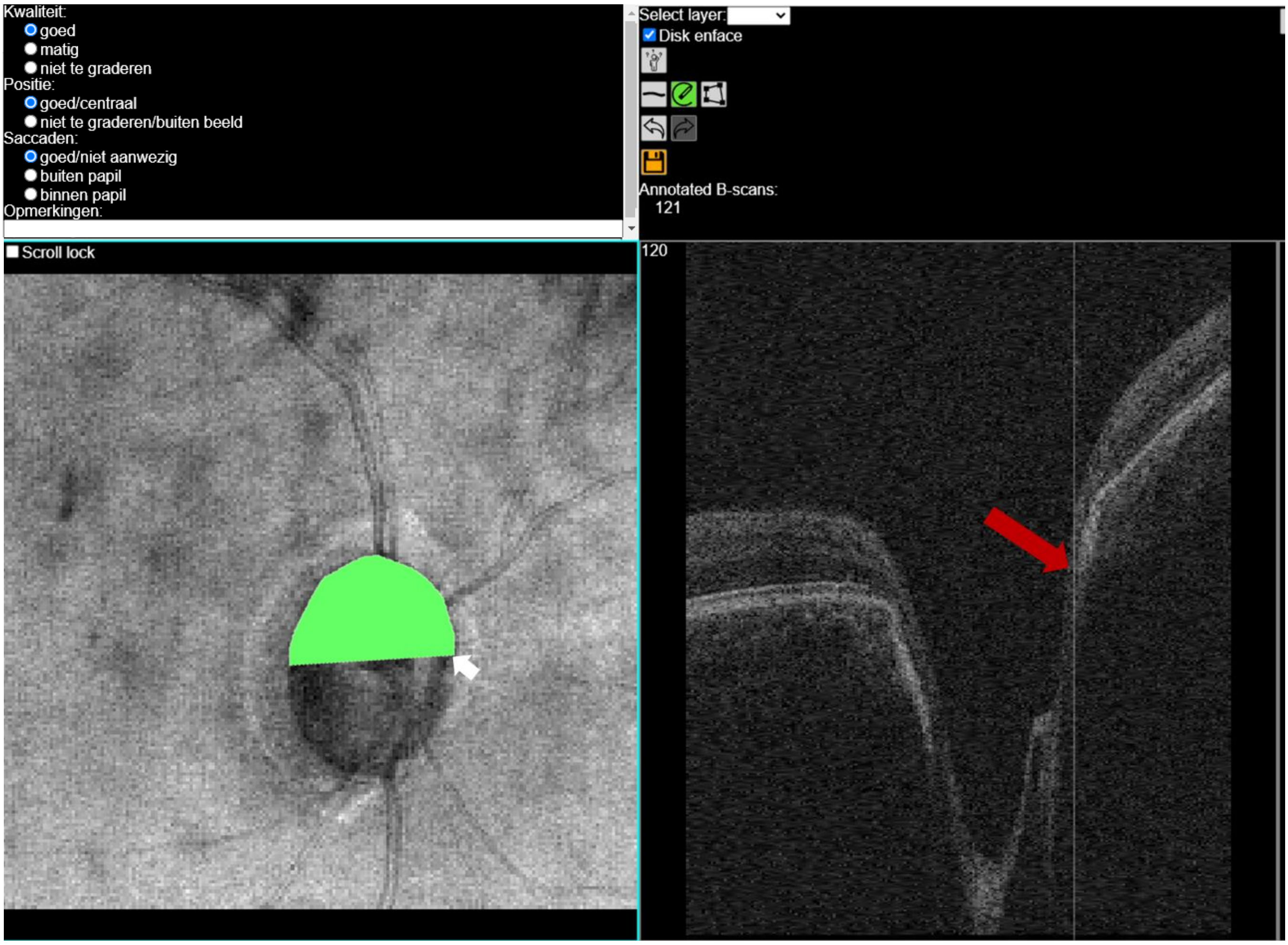

<u>Perform the following measurements (starting from step 6) for 5 B-scans per eye: one at the widest point of the optic nerve, two randomly above, and two randomly below. For these last four, also include B-scans outside the optic disc. The exception to this are the Spectralis scans. Here you also take 5 B-scans per eye: one horizontal, one vertical, two diagonal, and one circular scan (any one of the three).</u>

6. Retinal Nerve Fiber Layer (RNFL)

Draw the entire RNFL.

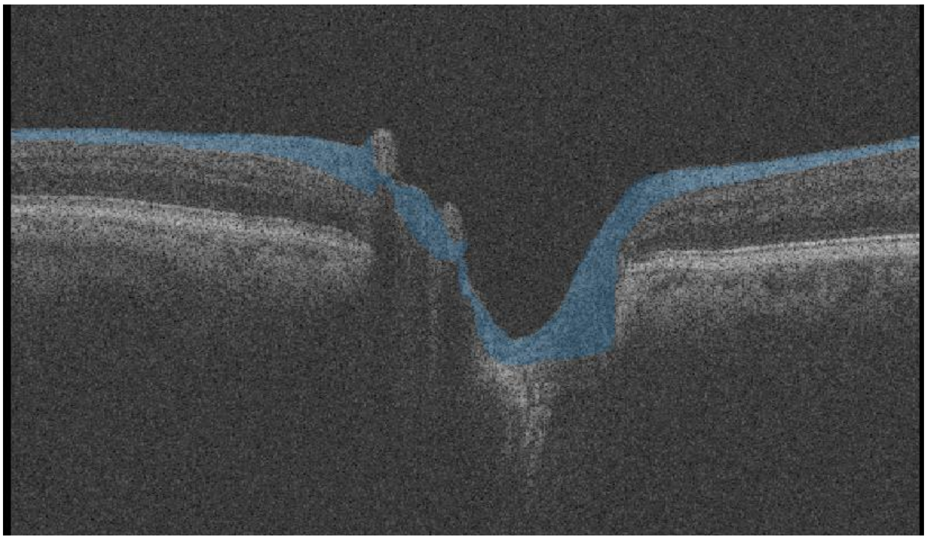

In some B-scans, the RNFL may not be easily distinguishable from other retinal layers. It can help to adjust the brightness and contrast (ctrl key and drag), or scroll through different B-scans until you find one where the RNFL is clear to get an indication of where the layer should be. If you are unsure whether you are dealing with the RNFL or the GCL, pay close attention to the INL. The INL is almost always clearly visible; if you reach this layer, you know you have gone too far (there is always GCL between the RNFL and INL).
If none of this works, you can mark the outlined area with the “uncertainty tool,” for example, at the “shoulder of the optic disc” where the RNFL transitions into pre-laminar tissue. Make sure that the upper boundary of the outlined RNFL (i.e., the ILM) is not marked as uncertain, as this is generally always visible.

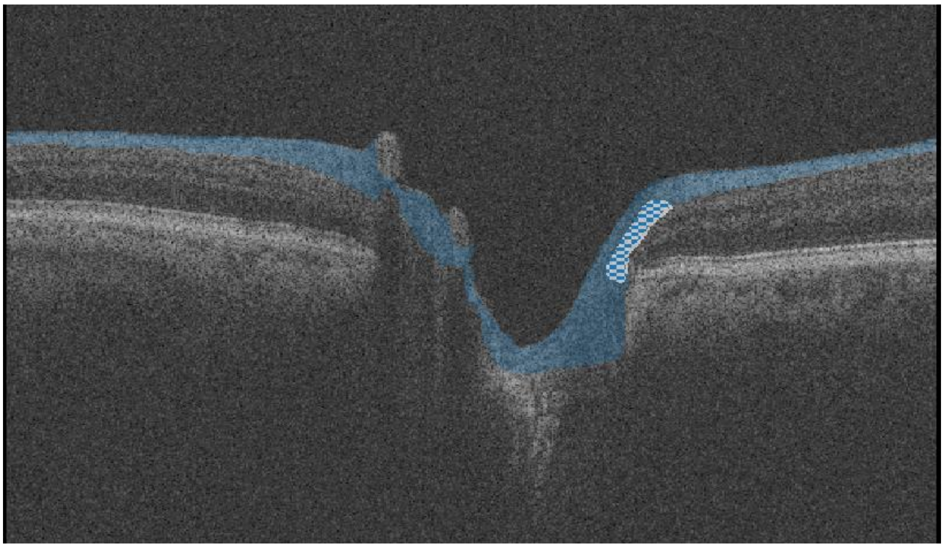

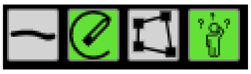

Do not use this tool to draw in an area where the signal does not penetrate; only draw what is visible.

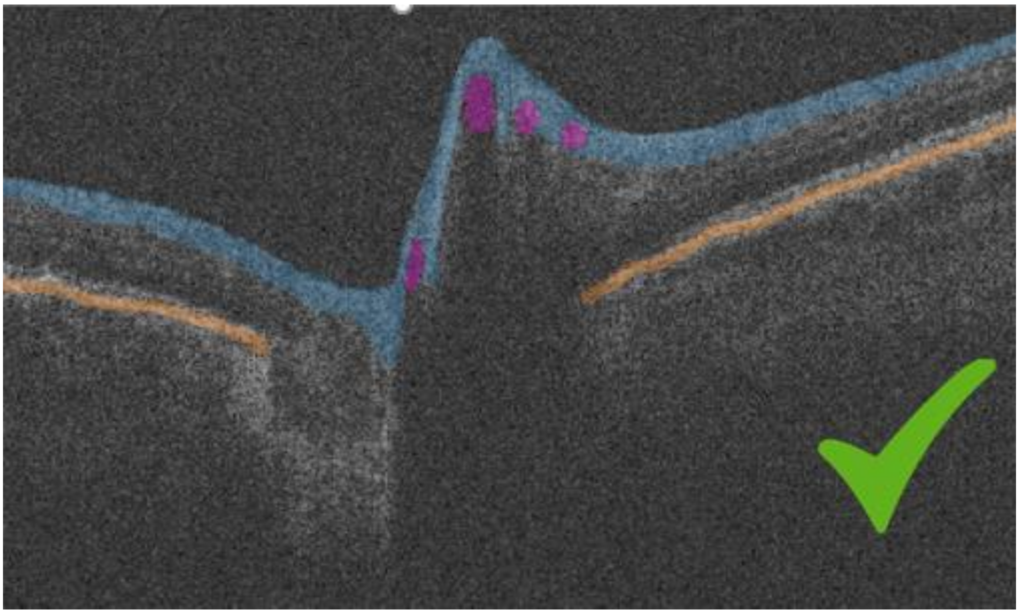

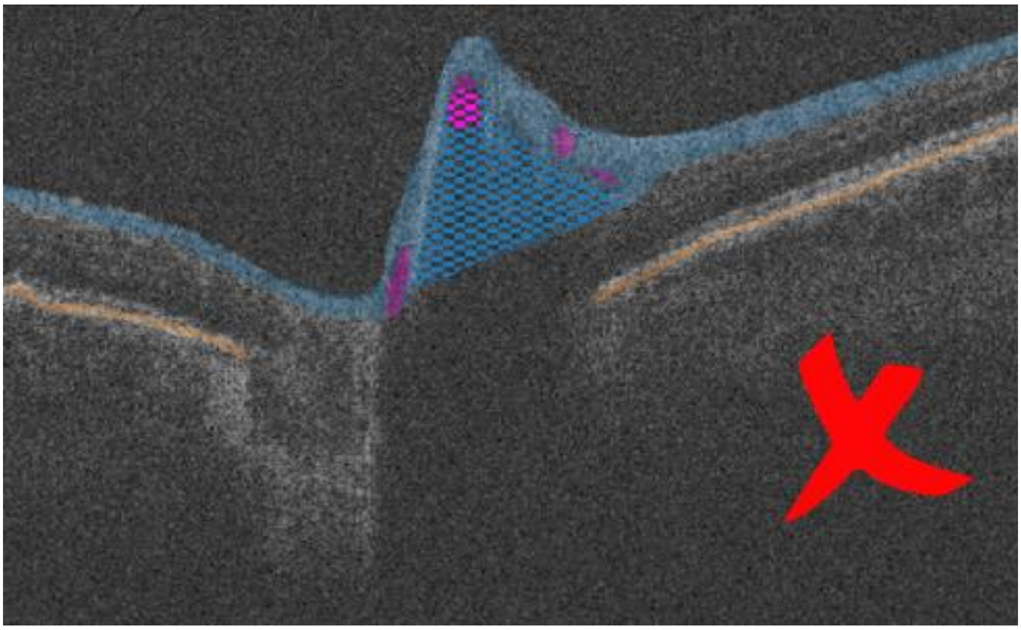

7. Bruch’s Membrane (BM) and peri papillary atrophy (PPA)

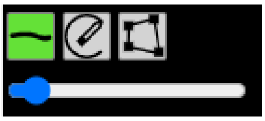

Draw the entire Bruch's membrane (actually, this is the BM/RPE complex). It's best to use the "line tool" for this. Adjust the thickness of the line with the slider so that it best matches the thickness of the BM. Make good use of the cursor on the en-face projection as well.

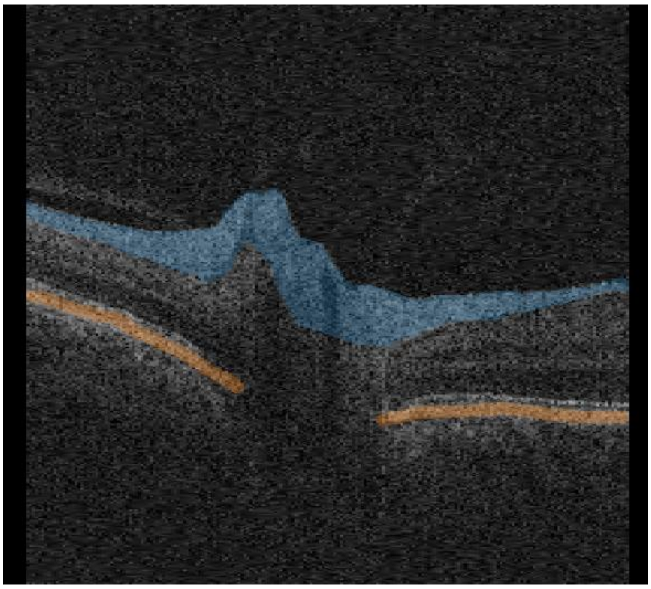

If there is peri-papillary atrophy, draw it based on the α/β/γ classification. In practice, α-PPA is often not easily distinguishable from β-PPA on lower quality OCT images. In such cases, draw it as β-PPA. The en-face projection can also be very helpful here (see examples on next page).

Example of β-zone PPA:

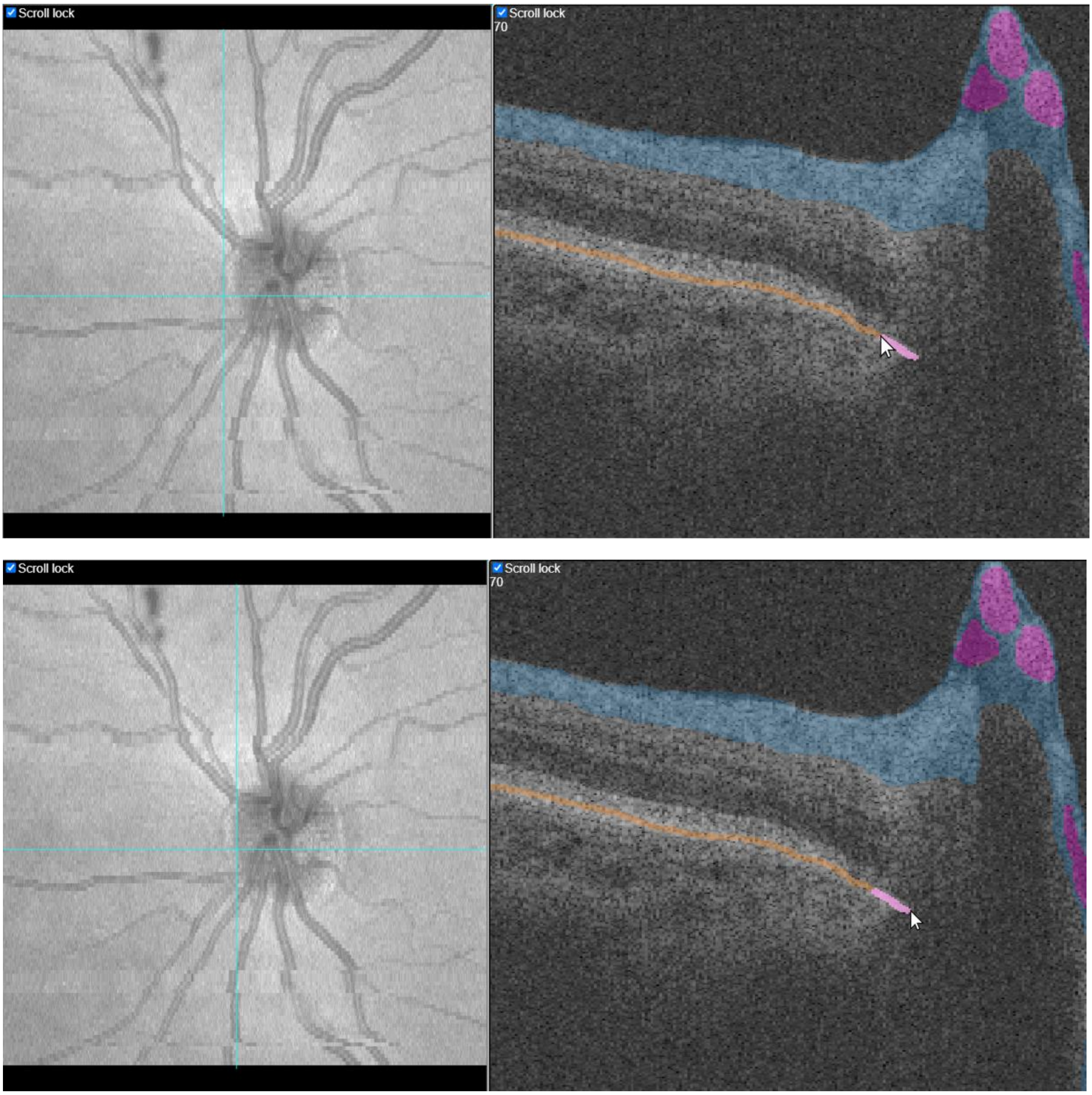

The hyperintensity of the transmitted signal below can be an indication of the absence of RPE, and thus the presence of β-PPA.

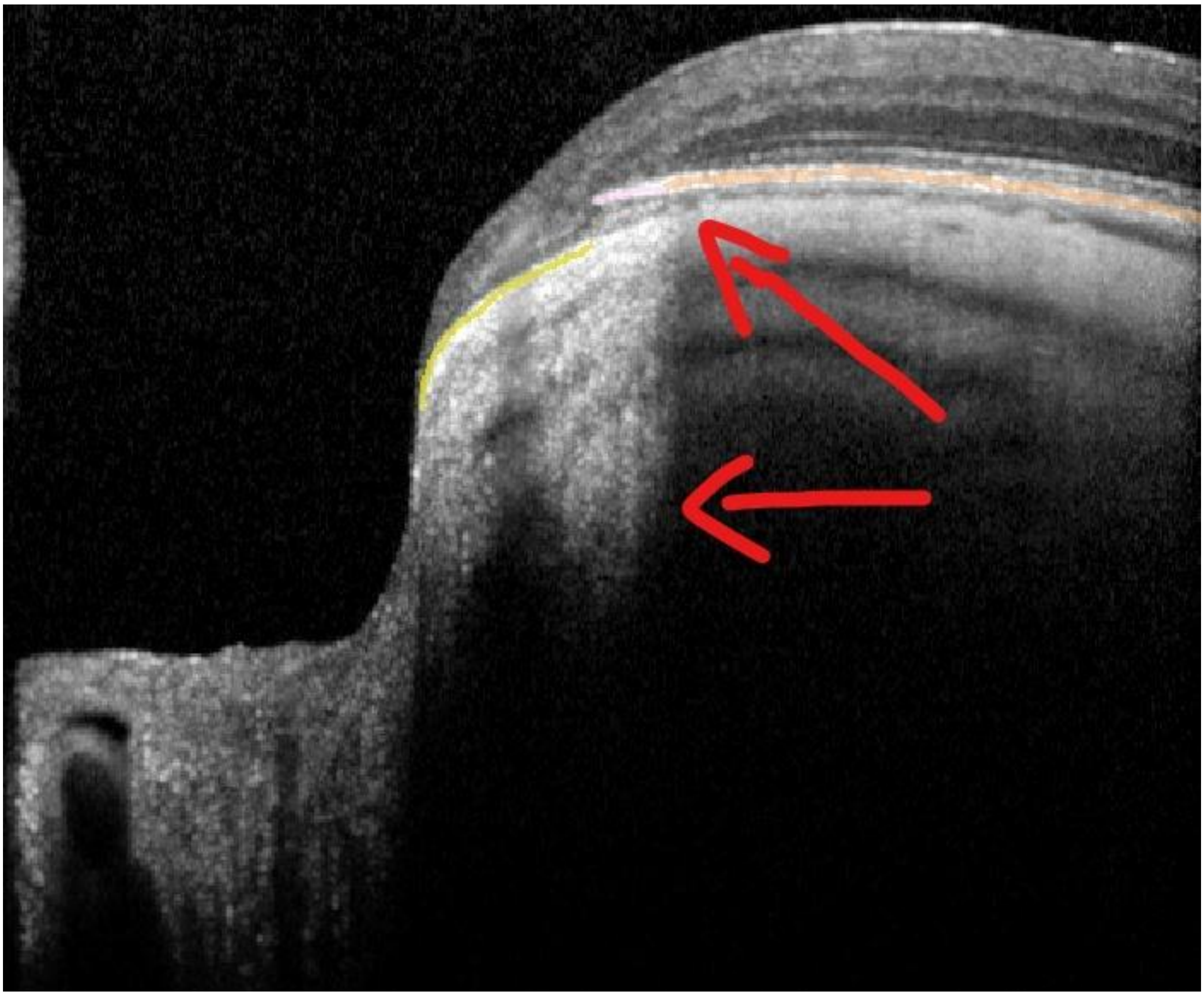

Example of γ-zone PPA:

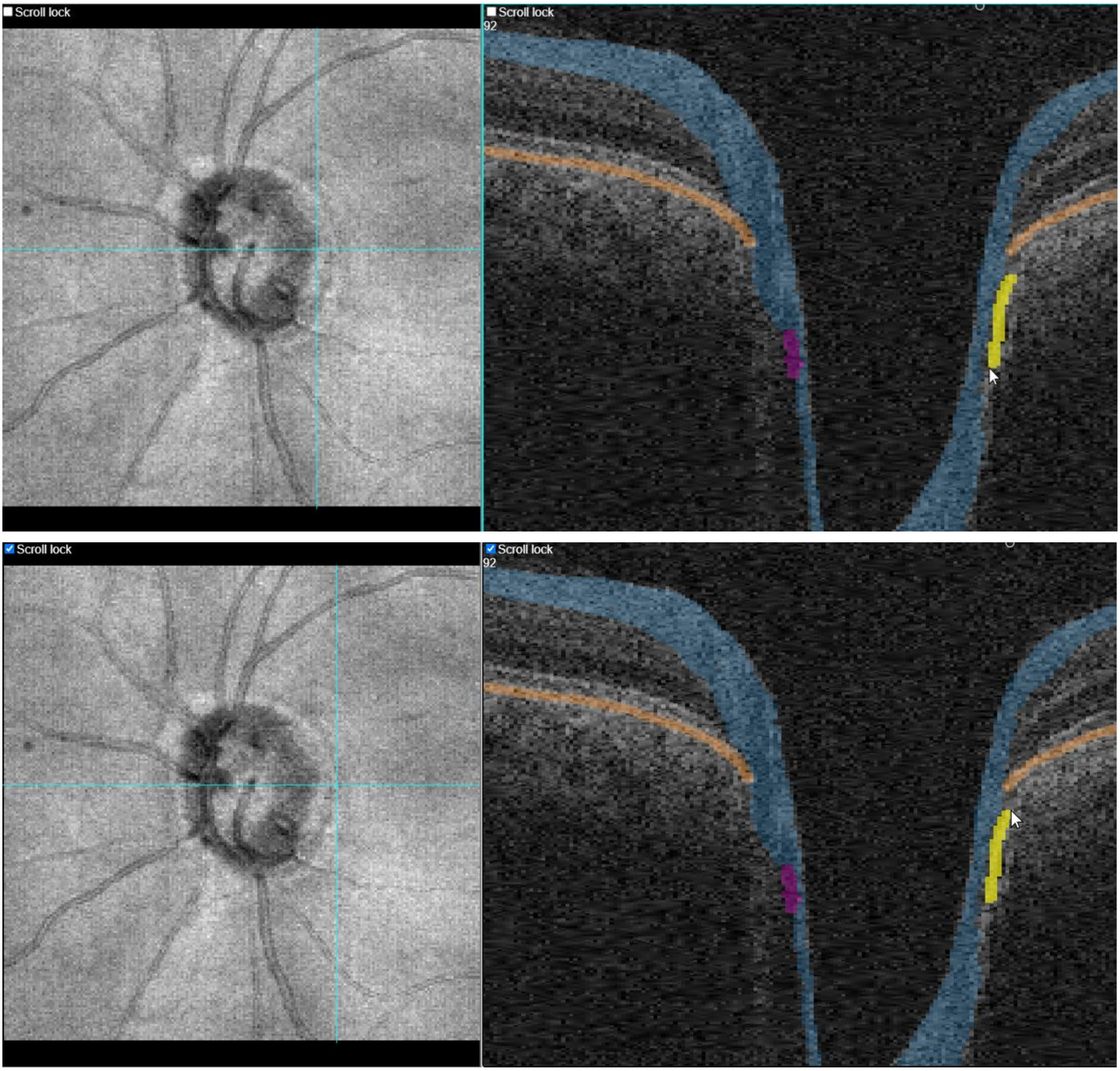

8. Lamina Cribrosa (LC)

Draw the upper edge of the lamina cribrosa. In principle, the LC directly connects to the drawn RNFL tissue.

In some B-scans, the LC may not be clearly visible or not visible at all. The "invert" option can sometimes help make the LC more visible. If you are unsure whether you have accurately annotated the LC, you can mark the drawn area with the "uncertainty tool." If the LC is not visible at all, skip step 8 entirely.

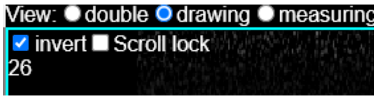

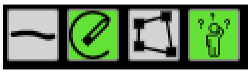

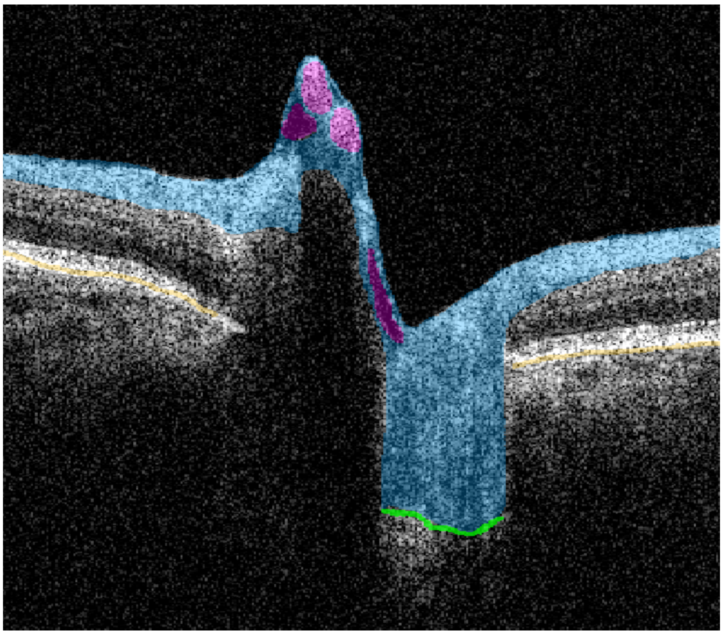

9. Blood vessels.

In many B-scans, you will also encounter blood vessels, often in the drawn RNFL and a portion of the GCL. Draw these blood vessels. The shadow that you often see below a blood vessel does not need to be drawn but can be a good marker for the presence of a vessel. If you are unsure whether you see a blood vessel on the B-scan, you can double-check this on the en-face projection (this also has an invert option, which often makes the vessels clearer). Note that the radial scans from Heidelberg can also cut through vessels lengthwise.

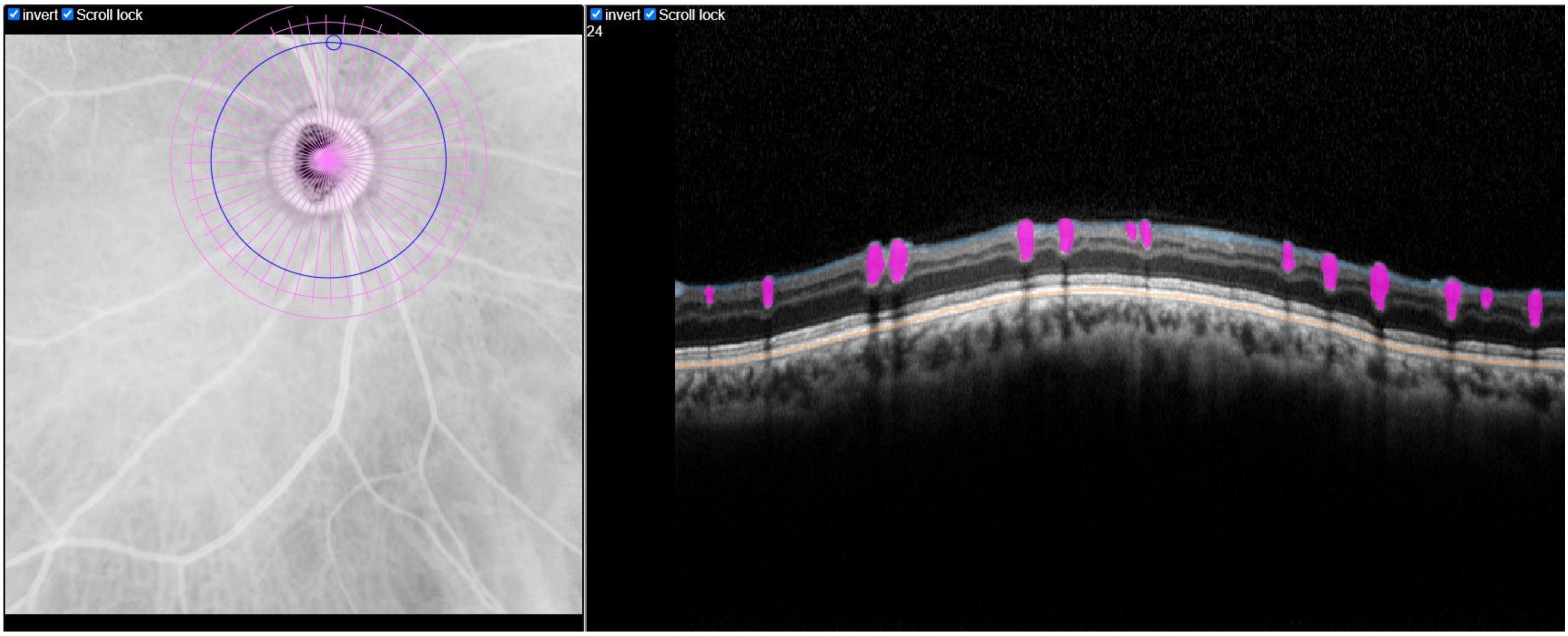

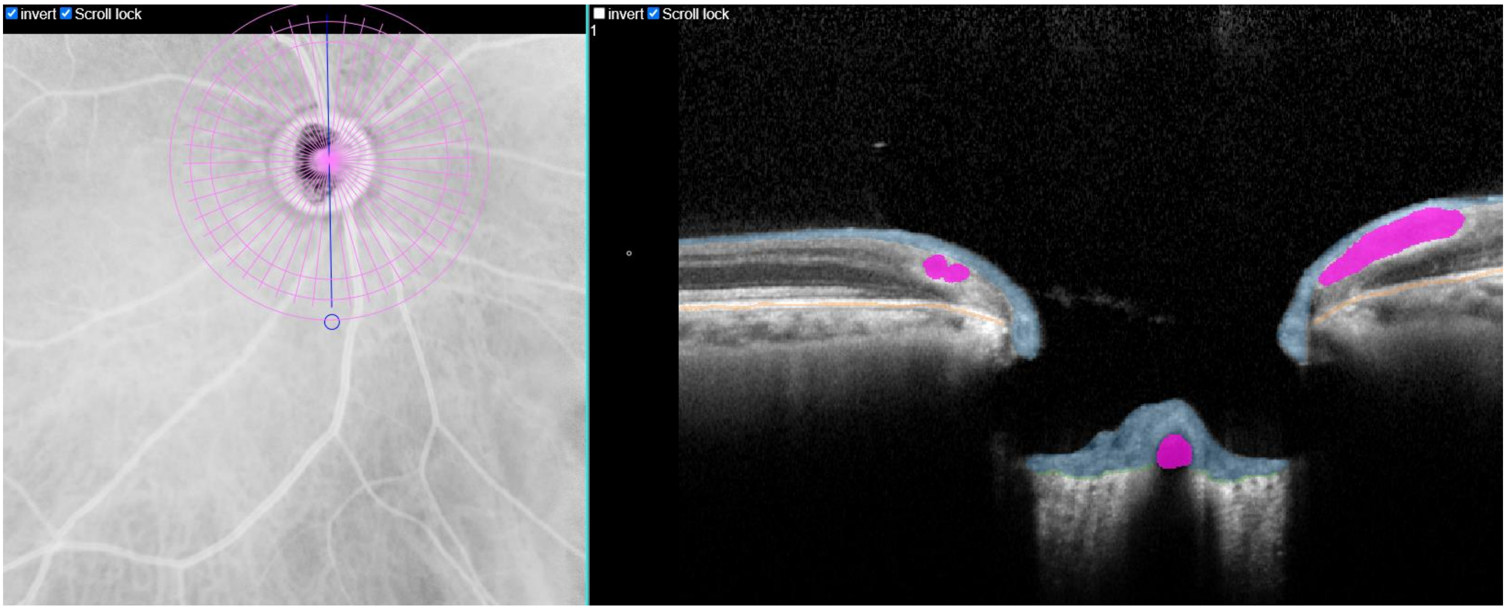

10. Landmarks.

If you are not working in "double view," first click the "measuring" checkbox.

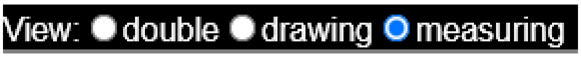

If you are working in "double view," perform this step in the rightmost window. Then click on the "Measure Landmarks" icon. This allows you to indicate the following structures in all B-scans at the location of the optic disc::

- Bruch’s Membrane (BM) left
- Bruch’s Membrane (BM) right
- Inner Limiting Membrane (ILM)
- Lamina Cribrosa (LC) top

If the LC is not visible, skip this step (**right-click**).

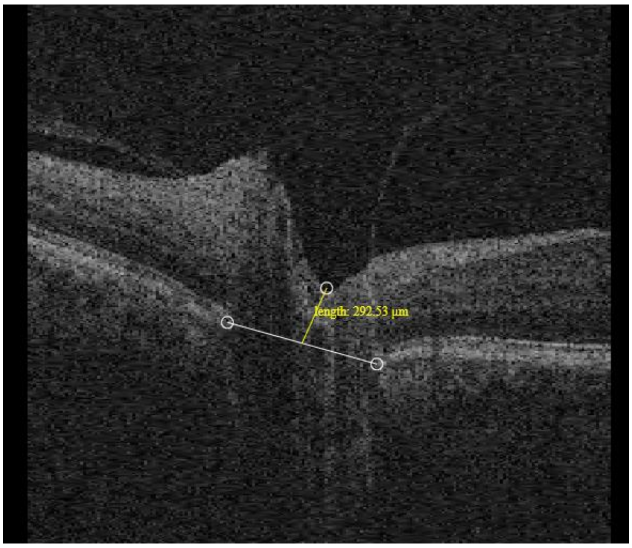

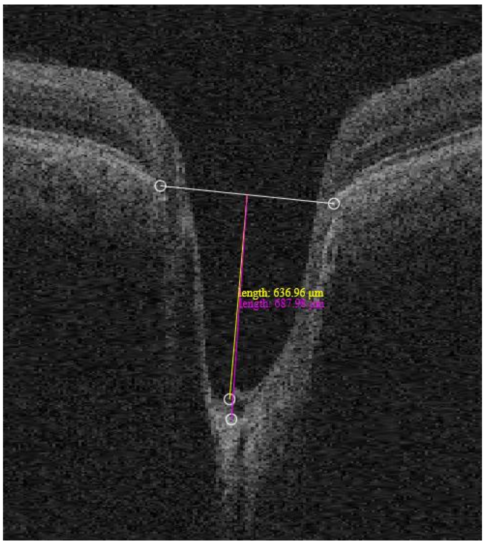

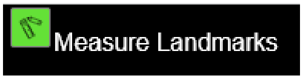

If you make a mistake when indicating the "landmarks," complete the measurement and then click "Measure Landmarks" again. This will overwrite the previously drawn points.
Be mindful of any γ-zone PPA; do not draw BM left and BM right on the scleral band, but rather at the outermost point of Bruch's membrane..

11. Save

When you have finished the annotations, press the "save" button and close the tab. 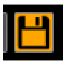

Supplement: Supplement 1 [file tvst-13-12-5_s001.pdf]
