# Supplementary material for: CNN-Based Device-Agnostic Feature Extraction From ONH OCT Scans
Source: Transl Vis Sci Technol. 2024 Dec 3;13(12):5. doi: 10.1167/tvst.13.12.5 (PMC11620008; doi:10.1167/tvst.13.12.5)
Supplement: Supplement 2 [file tvst-13-12-5_s002.pdf]

Supplementary Table S1: Validation performance of the model separated by device. Dice Similarity Coefficient (DSC) during validation in 5-fold cross-validation. Note that validation performance was used as criterion for early stopping of the model training.

	**Overall**	**Cirrus**	**1000 MKI**	**1000 MKII**	**3D-OCT 2000**	**Spectralis**
	Mean DSC ± SD	Mean DSC ± SD	Mean DSC ± SD	Mean DSC ± SD	Mean DSC ± SD	Mean DSC ± SD
RNFL	0.845 ± 0.08	0.779 ± 0.08	0.842 ± 0.06	0.846 ± 0.06	0.854 ± 0.05	0.891 ± 0.07
Vessels	0.483 ± 0.27	0.228 ± 0.20	0.496 ± 0.21	0.491 ± 0.17	0.454 ± 0.21	0.697 ± 0.20
Vessels + RNFL	0.870 ± 0.08	0.808 ± 0.09	0.863 ± 0.06	0.876 ± 0.06	0.887 ± 0.05	0.908 ± 0.07
Bruch's membrane[a]	0.575 ± 0.21	0.497 ± 0.15	0.488 ± 0.14	0.469 ± 0.13	0.427 ± 0.13	0.773 ± 0.15
BMO[b,c]	0.732 ± 0.40	0.659 ± 0.42	0.791 ± 0.37	0.669 ± 0.43	0.644 ± 0.43	0.863 ± 0.30
Lamina Cribrosa[b]	0.176 ± 0.22	0.138 ± 0.21	0.127 ± 0.12	0.062 ± 0.12	0.075 ± 0.12	0.249 ± 0.24
PPA Alpha[b]	0.036 ± 0.11	0.012 ± 0.07	0.005 ± 0.01	0.006 ± 0.02	0.069 ± 0.13	0.039 ± 0.12
PPA Beta[b]	0.238 ± 0.26	0.158 ± 0.19	0.094 ± 0.16	0.155 ± 0.17	0.135 ± 0.19	0.369 ± 0.30
PPA Gamma[b]	0.069 ± 0.16	0.077 ± 0.15	0.000 ± 0.00	0.023 ± 0.07	0.026 ± 0.07	0.105 ± 0.22

[a] Including PPA Alpha and PPA Beta. [b] If label present in either of the annotations. [c] Bruch's Membrane Opening, defined by absence of Bruch's membrane on enface projection

Supplementary Table S2: Concordance between grader segmentations and model in one-to-one comparisons of all grader pairs, and the model versus each grader. Dice Similarity Coefficients (DSC) is averaged over all annotated B-scans. A total of 40 B-scans were compared for each device (8 eyes, 5 B-scans per eye) annotated by 4 different graders.

	**Cirrus**		**3D-OCT 2000**		**Spectralis**	
	Inter-grader	Model vs graders	Inter-grader	Model vs graders	Inter-grader	Model vs graders
	Mean DSC ± SD	Mean DSC ± SD	Mean DSC ± SD	Mean DSC ± SD	Mean DSC ± SD	Mean DSC ± SD
RNFL	0.759 ± 0.07	0.807 ± 0.06	0.807 ± 0.08	0.846 ± 0.06	0.792 ± 0.11	0.826 ± 0.08

Vessels	0.288 ± 0.17	0.175 ± 0.16	0.359 ± 0.20	0.370 ± 0.22	0.600 ± 0.14	0.582 ± 0.17
Vessels + RNFL	0.805 ± 0.08	0.846 ± 0.07	0.851 ± 0.07	0.882 ± 0.06	0.848 ± 0.10	0.862 ± 0.07
Bruch's membrane[a]	0.332 ± 0.17	0.438 ± 0.20	0.363 ± 0.16	0.463 ± 0.16	0.319 ± 0.19	0.456 ± 0.21
BMO[b,c]	0.670 ± 0.38	0.674 ± 0.36	0.720 ± 0.37	0.674 ± 0.40	0.901 ± 0.17	0.919 ± 0.12
Lamina Cribrosa[b]	0.086 ± 0.16	0.059 ± 0.12	0.108 ± 0.19	0.018 ± 0.06	0.118 ± 0.16	0.117 ± 0.15
PPA Alpha[b]	0.018 ± 0.08	0.001 ± 0.01	0.059 ± 0.16	0.031 ± 0.09	0.058 ± 0.13	0.008 ± 0.02
PPA Beta[b]	0.111 ± 0.18	0.011 ± 0.06	0.156 ± 0.20	0.134 ± 0.19	0.174 ± 0.21	0.165 ± 0.21
PPA Gamma[b]	0.023 ± 0.06	0.000 ± 0.00	0.038 ± 0.12	0.001 ± 0.00	0.129 ± 0.20	0.000 ± 0.00

[a] Including PPA Alpha and PPA Beta. [b] If label present in either of the annotations. [c] Bruch's Membrane Opening, defined by absence of Bruch's membrane on enface projection

Supplementary Table S3. Concordance of cpRNFL thickness measurements, comparing manufacturers proprietary output.

	**Manufacturers**		
	Cirrus vs. 3D-OCT 2000		Cirrus vs. 3D-OCT 2000 vs. Spectralis
	ICC (95% CI)	Mean of absolute differences ± SD (µm)	ICC (95% CI)
cpRNFL @ 3.4mm, mean	0.590 (-0.079 – 0.901)	8.16 ± 3.91	NA
12 hour quartile	0.770 (0.238 – 0.949)	7.13 ± 5.03	NA
3 hour quartile	0.427 (-0.155 – 0.837)	12.25 ± 6.00	NA
6 hour quartile	0.795 (0.053 – 0.960)	8.50 ± 6.05	NA
9 hour quartile	0.716 (0.113 – 0.936)	10.38 ± 4.27	NA
cpRNFL @ 3.5mm, mean	NA	NA	NA

12 hour quartile	NA	NA	NA
3 hour quartile	NA	NA	NA
6 hour quartile	NA	NA	NA
9 hour quartile	NA	NA	NA
Temporal-superior	NA	NA	NA
Nasal-superior	NA	NA	NA
Nasal	NA	NA	NA
Inferior-nasal	NA	NA	NA
Inferior-temporal	NA	NA	NA
temporal	NA	NA	NA

cpRNFL=circumpapillary retinal nerve fiber layer; ICC=intraclass correlation coefficient; CI=confidence interval; NA=not applicable

Supplementary Table S4. Concordance of cup volume and BMO surface measurements, comparing manufacturers proprietary output.

	**Manufacturers**						
	Cirrus vs 3D-OCT 2000		Cirrus vs. Spectralis		3D-OCT 2000 vs. Spectralis		Cirrus vs. 3D-OCT 2000 vs. Spectralis
	ICC (95% CI)	Mean of absolute differences ± SD (µm)	ICC (95% CI)	Mean of absolute differences ± SD (µm)	ICC (95% CI)	Mean of absolute differences ± SD (µm)	ICC (95% CI)
Cup volume [a]	0.993 (0.923 – 0.999)	0.010 ± 0.011[b]	NA	NA	NA	NA	NA

BMO surface	0.584 (-0.072 – 0.913)	0.291 ± 0.126[c]	0.576 (-0.193 – 0.900)	0.230 ± 0.154[c]	0.322 (-0.221 – 0.790)	0.299 ± 0.284[c]	0.487 (0.083 – 0.841)

BMO=Bruch’s membrane opening; CNN=convolutional neural network; ICC=intraclass correlation coefficient; CI=confidence interval; NA=not applicable; [a] 2 out of 8 eyes had no cup below the BMO plane, resulting in a value of 0. [b] Expressed in mm^3. [c] Expressed in mm^2.

Supplement table S5. Concordance of cpRNFL thickness measurements, comparing manufacturers proprietary output to the CNN output within devices.

	**Manufacturer vs. CNN**					
	Cirrus		3D-OCT 2000		Spectralis	
	ICC (95% CI)	Mean of absolute differences ± SD (µm)	ICC (95% CI)	Mean of absolute differences ± SD (µm)	ICC (95% CI)	Mean of absolute differences ± SD (µm)
cpRNFL @ 3.4mm, mean	0.815 (0.301 – 0.960)	4.62 ± 2.32	0.916 (0.668 – 0.982)	3.31 ± 2.30	NA	NA
12 hour quartile	0.531 (-0.286 – 0.888)	9.94 ± 6.00	0.678 (-0.082 – 0.932)	8.90 ± 6.48	NA	NA
3 hour quartile	0.777 (0.211 – 0.951)	5.61 ± 5.00	0.747 (0.155 – 0.944)	6.45 ± 6.86	NA	NA
6 hour quartile	0.971 (0.870 – 0.994)	3.07 ± 2.10	0.919 (0.642 – 0.983)	6.20 ± 2.51	NA	NA
9 hour quartile	0.897 (0.571 – 0.979)	4.48 ± 2.72	0.967 (0.847 – 0.993)	3.62 ± 2.55	NA	NA
cpRNFL @ 3.5mm, mean	NA	NA	NA	NA	0.873 (-0.038 – 0.981)	4.23 ± 1.83
12 hour quartile	NA	NA	NA	NA	NA	NA
3 hour quartile	NA	NA	NA	NA	NA	NA
6 hour quartile	NA	NA	NA	NA	NA	NA
9 hour quartile	NA	NA	NA	NA	NA	NA
Temporal-superior	NA	NA	NA	NA	0.914 (0.515 – 0.983)	6.77 ± 5.24
Nasal-superior	NA	NA	NA	NA	0.929 (0.703 – 0.985)	6.75 ± 5.13
Nasal	NA	NA	NA	NA	0.916 (-0.009 – 0.988)	6.86 ± 3.20
Inferior-nasal	NA	NA	NA	NA	0.972 (0.854 – 0.994)	5.75 ± 4.25
Inferior-temporal	NA	NA	NA	NA	0.812 (0.134 – 0.962)	7.65 ± 4.82
temporal	NA	NA	NA	NA	0.806 (-0.028 – 0.970)	8.01 ± 2.30

CNN=convolutional neural network; cpRNFL=circumpapillary retinal nerve fiber layer; ICC=intraclass correlation coefficient; CI=confidence interval; NA=not applicable

Supplementary Table S6. Concordance of BMO-MRW measurements, comparing manufacturers proprietary output to the CNN output within devices

	Manufacturer vs. CNN					
	Cirrus		3D-OCT 2000		Spectralis	
	ICC (95% CI)	Mean of absolute differences ± SD (μm)	ICC (95% CI)	Mean of absolute differences ± SD (μm)	ICC (95% CI)	Mean of absolute differences ± SD (μm)
BMO-MRW, mean	NA	NA	NA	NA	0.983 (0.917 – 0.997)	13.69 ± 8.79
12 hour quartile	NA	NA	NA	NA	NA	NA
3 hour quartile	NA	NA	NA	NA	NA	NA
6 hour quartile	NA	NA	NA	NA	NA	NA
9 hour quartile	NA	NA	NA	NA	NA	NA
Temporal-superior	NA	NA	NA	NA	0.717 (-0.047 – 0.942)	44.93 ± 32.22
Nasal-superior	NA	NA	NA	NA	0.709 (0.006 – 0.937)	58.17 ± 55.09
Nasal	NA	NA	NA	NA	0.986 (0.915 – 0.997)	14.19 ± 13.20
Inferior-nasal	NA	NA	NA	NA	0.571 (-0.047 – 0.891)	77.08 ± 84.66
Inferior-temporal	NA	NA	NA	NA	0.788 (0.169 – 0.955)	39.77 ± 27.46
temporal	NA	NA	NA	NA	0.968 (0.864 – 0.993)	15.62 ± 14.17
Cup volume [a]	0.680 (0.019 – 0.927)	0.057 ± 0.065[b]	0.713 (0.088 – 0.935)	0.046 ± 0.059[b]	NA	NA
BMO surface	0.136 (-0.163 – 0.622)	0.434 ± 0.322[c]	0.275 (-0.397 – 0.787)	0.251 ± 0.135[c]	0.483 (-0.133 – 0.861)	0.204 ± 0.217[c]

BMO=Bruch's membrane opening; MRW=minimal rim width; CNN=convolutional neural network; ICC=intraclass correlation coefficient; CI=confidence interval; NA=not applicable; [a] 2 out of 8 eyes had no cup below the BMO plane, resulting in a value of 0. [b] Expressed in mm^3. [c] Expressed in mm^2.

Supplementary Table S7. Concordance of cup volume and BMO surface measurements, comparing CNN output between manufacturers.

CNN

	Cirrus vs 3D-OCT 2000		Cirrus vs. Spectralis		3D-OCT 2000 vs. Spectralis		Cirrus vs. 3D-OCT 2000 vs. Spectralis
	ICC (95% CI)	Mean of absolute differences ± SD	ICC (95% CI)	Mean of absolute differences ± SD	ICC (95% CI)	Mean of absolute differences ± SD	ICC (95% CI)
Cup volume [a]	0.990 (0.950 – 0.998)	0.006 ± 0.006[b]	NA	NA	NA	NA	NA
BMO surface	-0.078 (-0.465 – 0.536)	0.277 ± 0.325[c]	-0.133 (-0.587 – 0.534)	0.278 ± 0.328[c]	0.953 (0.745 – 0.991)	0.049 ± 0.020[c]	0.062 (-0.191 – 0.552)

BMO=Bruch's membrane opening; CNN=convolutional neural network; ICC=intraclass correlation coefficient; CI=confidence interval; NA=not applicable; [a] 2 out of 8 eyes had no cup below the BMO plane, resulting in a value of 0. [b] Expressed in mm^3. [c] Expressed in mm^2.
